# Light at night exposure and risk of breast cancer: a meta-analysis of observational studies

**DOI:** 10.3389/fpubh.2023.1276290

**Published:** 2023-12-01

**Authors:** Zining Luo, Zhenglong Liu, Hongjie Chen, Ying Liu, Nenghuan Tang, Haoran Li

**Affiliations:** Department of Stomatology, North Sichuan Medical College, Nanchong, China

**Keywords:** breast cancer, light at night, lighting, menopausal status, meta-analysis

## Abstract

**Objective:**

The aim of this meta-analysis is to evaluate the impact of light at night (LAN) exposure on the risk of breast cancer across varying factors.

**Method:**

We conducted a systematic search of literature up to July 15, 2023, including PubMed, Cochrane Library, and Embase databases, using keywords related to breast cancer and LAN exposure. Cohort study and case–control study literature on night light exposure and breast cancer risk were included. Statistical analyses were performed using Stata software version 17.0. To address heterogeneity among different studies, we employed a random-effects model for analysis and assessed publication bias using funnel plots and Egger’s test.

**Results:**

We included 13 case–control and 8 cohort studies with 734,372 participants worldwide. In the Newcastle-Ottawa Scale (NOS) assessments, the average score was 7.43 (ranging from 5 to 9). The overall meta-analysis demonstrated a significant association between exposure to LAN and risk of breast cancer (RR = 1.12; 95% CI: 1.06–1.17; I^2^ = 31.3%, *p* < 0.001). In the subgroup analysis, the results of the analysis for study types (case–control studies: RR = 1.16; 95% CI: 1.06–1.27; I^2^ = 40.4%, *p* = 0.001; cohort studies: RR = 1.08; 95% CI: 1.04–1.14; I^2^ = 0.0%, *p* < 0.001) and the results for light exposure types (outdoor LAN: RR = 1.07; 95% CI: 1.02–1.13; I^2^ = 30.9%, *p* = 0.004) are presented. In the analysis conducted for continents, the highest breast cancer risk was observed in the Asian population (Asian: RR = 1.24; 95% CI: 1.15–1.34; I^2^ = 0.0%, *p* < 0.001) and in the analysis of estrogen receptor status (ER+: RR = 1.10; 95% CI: 1.03–1.18; I^2^ = 17.0%, *p* = 0.005;). We also conducted an analysis on menopausal status and various lifestyles but did not find any statistically significant findings.

**Conclusion:**

Our study demonstrates that LAN exposure is associated with an increased risk of breast cancer, particularly in the Asian population. Among the existing hypotheses, the idea that LAN exposure leads to a decrease in melatonin is widely accepted. However, until the mechanism of this effect is clearly elucidated, it is not recommended to take melatonin supplements for breast cancer prevention without medical advice. We hope to conduct more high-quality research, especially concerning the investigation of other environmental confounding factors, to further advance this field.

## 1 Introduction

Breast cancer is a prevalent form of cancer among women worldwide. The incidence and mortality of breast cancer have increased among women worldwide (1, 2). In 2018, the number of breast cancer cases in Italy increased by 12.5% compared to the prevalence in 2012 (3). Within North American countries, the incidence of breast cancer increased from 29% in 1987 to 70% in 2000 (4). Despite a decrease in the use of menopausal hormone therapy from 2000 to 2004 due to the influence of the Women’s Health Initiative (5, 6), the incidence of invasive breast cancer decreased (6, 7). However, since 2004, the incidence of invasive breast cancer has been slowly rising at a rate of 0.5% per year (8–10). Globally, the annual number of newly diagnosed breast cancer cases has exceeded 11.6% (11).

Breast cancer, as a highly heterogeneous and multifactorial disease (12), has been the subject of extensive research since the beginning of the 21st century (13). Efforts have been made to identify the associated risk factors and reduce the incidence of breast cancer in women. Current lifestyle risk factors for breast cancer primarily include factors such as ethnicity (14), geographical location (15), occupation (16), dietary choices (17), air quality levels (18), national economic development (19), smoking habits (20), alcohol consumption (21), and hormonal factors (22). Additionally, recent ecological research has identified an increase in body mass index (BMI) and a sustained decrease in birth rates as potential risk factors for an increased risk of breast cancer (23–25). In terms of environmental factors, an association has been observed between increased breast cancer risk and light pollution (26).

Light pollution is an emerging environmental issue that has intensified with urbanization and industrialization. Over the past few decades, light pollution has posed an increasing threat to human health (27). Between 2012 and 2016, artificial light at night (LAN) on Earth increased by 2.2% annually, with a total annual increase in radiance of 1.8% (28). According to the “New World Atlas of Artificial Sky Brightness,” more than 80% of the world’s population and over 99% of the population in the United States and Europe live under light-polluted skies (29). Outdoor LANs, such as urban artificial lighting, can infiltrate indoor environments, although the relationship between outdoor and indoor LAN exposure is not yet fully understood (30). Additionally, indoor LAN exposure has increased in recent decades, primarily due to household lights left on at night and new sources of exposure, such as screens and electronic devices such as smartphones, which have polluted the natural darkness of the night (31). In recent years, a substantial body of epidemiological evidence, including over 20 studies (32–52), has investigated the relationship between exposure to outdoor LAN (32, 39, 41, 45–48, 52), measured through satellite measurements in specific study areas, and breast cancer incidence. Self-reported indoor LAN exposure has also been studied (33, 35, 37, 43, 51). This evidence has identified LAN exposure as a risk factor for breast cancer.

Existing hypotheses suggest that exposure to LAN may inhibit the natural surge of melatonin during the night (53, 54), thereby diminishing the antitumor proliferative capacity or elevating circulating levels of estrogen and progesterone (55). Estrogen is a significant risk factor for breast cancer (56). Such an impact may lead to an increased susceptibility to breast cancer.

A series of epidemiological studies have examined the impact of LAN exposure on breast cancer risk, considering factors such as classification of this exposure, hormone receptor status, race, menopausal status, and others. In various studies examining different types of LAN exposure, previous research has reported a positive association between outdoor LAN exposure and breast cancer risk (46, 47). However, the results regarding self-reported indoor LAN exposure have been inconsistent. Specifically, in premenopausal women, some studies have suggested that LAN exposure increases the risk of breast cancer (46), while others have not observed any statistically significant associations (36). Furthermore, in analyses considering hormone receptor status, existing research has noted that LAN exposure has a significant promoting effect on estrogen receptor-positive breast cancer and in white populations. As this research area continues to receive increasing attention, new epidemiological studies are still ongoing to further investigate these relationships (33, 34, 41).

In the past 2 years, a recent meta-analysis included an analysis of BMI and reanalyzed the impact of breast cancer risk in different estrogen receptor status populations (57). Additionally, four new large-scale studies on nocturnal light exposure have drawn attention to research related to pregnancy hormone receptors, ethnicity, and population characteristics (37, 41, 45, 47). However, previous meta-analyses have overlooked the potential influence of nocturnal light and lifestyle factors, and they did not investigate the status of progesterone receptors, race, or other factors that are currently of interest in the field. Considering the potential impact of different races, regions, and progesterone receptor factors on breast cancer, we have included several recently published studies, updating the meta-analysis in this field. We have comprehensively re-evaluated the multifaceted associations between local area network exposure and breast cancer.

## 2 Methods

### 2.1 Study protocol

The present meta-analysis was performed in accordance with the Preferred Reporting Items for Systematic Reviews and Meta-Analyses [PRISMA; 58] guidelines for systematic reviews and meta-analysis. Our protocol has been registered on the PROSPERO platform under registration number CRD42023446724.

### 2.2 Search approach

We searched the PubMed, Cochrane Library, and Embase databases up to July 15, 2023. We employed Medical Subject Headings (MeSH) and relevant keywords. We drew upon the previous methodologies of meta-analysis. (57, 59–64). We included case–control and cohort studies without any language restrictions. The search terms included “Breast Neoplasms,” “Malignant Neoplasm of Breast,” “Light exposure,” and “Risk.” The complete search strategy for each database can be found in Supplementary Tables 1–3. Additionally, we scrutinized the reference lists of the included cohort studies and other published meta-analyses to identify relevant observational studies.

### 2.3 Study selection

The study selection process was carried out by two reviewers (LZN and LZL) in accordance with the predetermined inclusion and exclusion criteria. Both reviewers independently screened the literature and removed duplicate and irrelevant articles based on titles and abstracts. Subsequently, the full texts of potentially eligible articles were obtained and thoroughly reviewed to ensure the inclusion of all relevant studies. Any disagreements between the two reviewers were resolved through discussion until a consensus was reached.

### 2.4 Eligibility criteria

The inclusion criteria were as follows: (a) inclusion of an independent exposure variable pertaining to LAN exposure; (b) consideration of an outcome variable related to breast cancer; (c) incorporation of case–control or cohort study designs. The exclusion criteria were as follows: (a) reviews, conference proceedings, or commentaries; (b) duplicate literature (retaining the variant with the most comprehensive information).

### 2.5 Data extraction

The data extraction process was conducted independently by the first reviewer (LZN), and upon its completion, cross-verification and consultation was performed with the second reviewer (LZL). A predesigned data extraction form was utilized, and the following data were extracted: study type (case–control or cohort), first author, publication year, source of cases, source of controls, number of cases, number of controls, exposure definition, breast cancer definition, classification of exposure, information collection period and scope, baseline age, and values of outcome variables (odds ratio or hazard ratio) determined based on various confounding factors. Any potential discrepancies were addressed through discussions with LZL to achieve a consensus.

### 2.6 Quality assessment

The quality of our studies was evaluated using the Newcastle-Ottawa Scale (NOS) (65), which employs a star system ranging from 0 to 9 to evaluate participant selection and exposure measurement, with 2 stars allocated for result comparison and 3 stars designated for outcome assessment and follow-up adequacy. A higher score reflects superior study quality. Scores ranging from 0 to 3 indicate low quality, scores ranging from 4 to 6 indicate moderate quality, and scores ranging from 7 to 9 indicate high quality.

### 2.7 Statistical analysis

The adjusted relative ratios (RRs) and 95% confidence intervals (CIs) for each observational study were used to assess the association between breast cancer incidence and LAN exposure. Heterogeneity was evaluated using I^2^ values (66) and Cochran’s Q heterogeneity test (67). Furthermore, we utilized Z-tests to assess whether the effect size (RRs) is equal among different subgroups (67). Based on the precedent of previous meta-analyses (33–35), we used a random effects model in each study to address confounding factors and minimize study errors. Sensitivity analysis was conducted by predefining the exclusion of studies that did not pass the sensitivity analysis to ensure the robustness of the overall effect. A funnel plot was examined for symmetry to detect publication bias, and Egger’s regression test was used for statistical assessment (68). The primary data analysis was conducted using Stata statistical software version 17.0 (Stata Corp, College Station, Texas), while the Z-tests were performed using R language (version 4.2.2).

## 3 Results

### 3.1 Literature search

**Figure 1:**
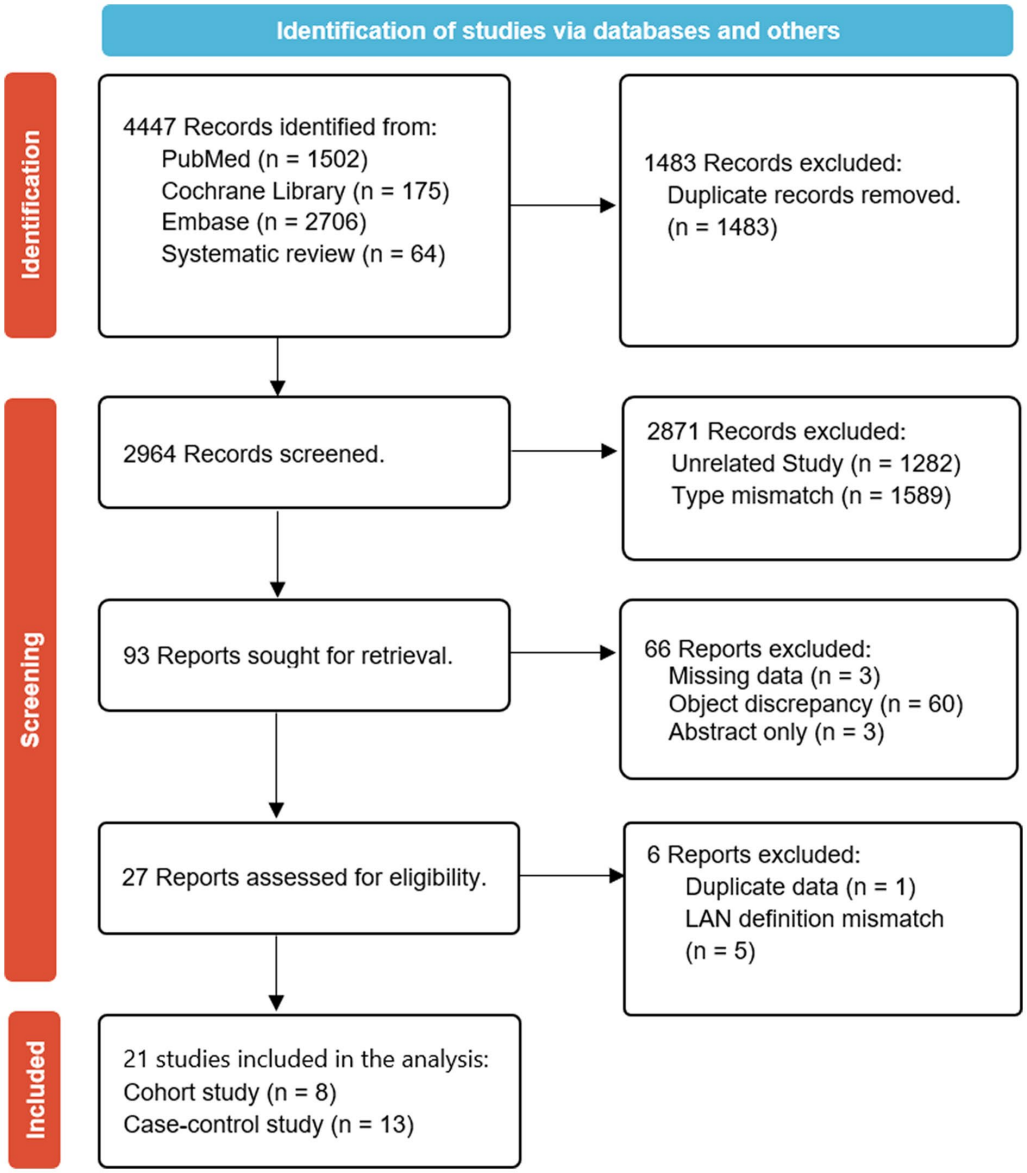
Preferred reporting items for systematic reviews and meta-analysis (PRISMA) flow diagram summarizing the search strategy and study selection for the meta-analysis of the association between LAN exposure and risk of breast cancer.

A total of 2,964 observational studies were initially identified after excluding duplicates. After conducting title and abstract screening, 2,871 articles were subsequently excluded. An in-depth review was conducted on 93 articles, and after the further exclusion of 72 articles, 21 studies were eligible for the meta-analysis (Figure 1). The primary reasons for exclusion were irrelevant research topics, inappropriate study types, missing data, inappropriate study subjects, availability of only abstracts, studies sharing the same data, and unclear definitions within the LAN. Among the 21 included studies, 8 were cohort studies (36, 39, 41, 45–47, 50, 52) and 13 were case–control studies (32–35, 37, 38, 40, 42–44, 48, 49, 51).

### 3.2 Study characteristics

The 21 studies included in this study were published between 2001 and 2023 and involved a total of 734,372 participants. Of these studies, 11 originated from North America (32, 39–41, 43, 46–50, 52), 3 from Europe (36, 37, 45), 5 from Asia (33–35, 38, 42), and an additional 2 from Australia (44, 51). The average follow-up time for these studies was 12.8 years.

Among the various confounding factors examined in these studies, the proportion of studies focused on estrogen receptor status was 42.9% (*n* = 9) (36, 37, 41, 42, 45–47, 50, 52), while studies on menopausal status accounted for 47.6% (*n* = 10) (36, 39–42, 45–48, 51). Additionally, studies investigating indoor LAN accounted for 66.7% (*n* = 14) (33–44, 50, 51), and studies on outdoor LAN accounted for 66.7% (*n* = 14) (32–35, 37, 39–41, 45–48, 50, 52).

Regarding lifestyle habits, the proportions were as follows: 19.0% (*n* = 4) of studies examined the habit of watching TV while sleeping (35, 40, 41, 50), 23.8% (*n* = 5) looked at the use of bedroom shutters (33–35, 38, 40), and 14.3% (*n* = 3) explored the habit of waking up and turning on lights (36, 43, 49).

Each study provided clear definitions for both LAN exposure and breast cancer. Adjusted estimates were available for analysis, despite the presence of varying confounding factors across the studies. The characteristics of the cohort (Table 1) and case–control studies (Table 2) are presented.

**Table 1:** Summary of main characteristics of the eight cohort studies examining association between exposure to LAN and risk of breast cancer.

Author (year)	Setting	Period	Analysis sample	LAN assessment	Breast cancer (definition)	Age at baseline	Follow-up year
Johns et al. (2018) (36)	UK	2003–2012	105,866	Self-reported level of exposure to LAN within the year prior to recruitment and at the age of 20 years (in the room where the subjects slept).	Simultaneously includes invasive breast cancer and ductal carcinoma *in situ* (DCIS).	16–102	6.1 years
James et al. (2017) (46)	USA	1989–2013	109,672	(Calculated cumulative average outdoor LAN for each participant at each questionnaire response) The data on annual outdoor LAN was derived from satellite imagery obtained from the U.S. DMSP’s Operational Linescan System	invasive breast cancer (As per ICD-8 code 174.0 – Malignant neoplasm of breast)	25–42	Over 22 years
White et al. (2017) (50)	USA	2003–2009	50,533	The self-report form of LAN assessment method was used during data collection and follow-up, which includes information about the type of lighting and whether the lights are turned on when waking up at night.	Simultaneously includes invasive breast cancer and ductal carcinoma *in situ* (DCIS).	35–74	7.4 years
Hurley et al. (2015) (39)	USA	1995–2001	106,731	Indoor LAN refers to the self-reported exposure to LAN (artificial light at night) at home while sleeping during the one-year period before the baseline survey. Outdoor LAN refers to the exposure to LAN assessed at the baseline survey, specifically around the geocoded residential address. This data is modeled from DMSP (Defense Meteorological Satellite Program) data.	incident invasive carcinoma of the breast (as per SEER site code:26000)	22–104	NA
Xiao Q. et al. (2021) (52)	USA	2002–2017	43,500	The LAN area level corresponds to the business line scanning system using geodetic baseline addresses based on self-reporting (2002–2009) and the 2014 US Defense Weather Satellite Program.	NA	NA	Over 11 years
Clarke R. B. et al. (2021) (45)	Danish	1993–1999	16,941	Based on self-reported and organized address information, outdoor LAN data is obtained according to DMSP correspondences.	primary invasive breast cancer	44–104	17.1 years
Xiao Q. et al. (2020) (47)	USA	1995–1996	186,981	Based on self-reported and organized address information [ArcGIS (v. 10.7, ESRI, Redlands, California)], outdoor LAN data is obtained according to DMSP correspondences.	Various grades, forms, and stages of breast cancer are included (*In situ*, local, regional/remote are included)	NA	15 years
Marina et al. (2022) (41)	USA	2003–2009	47,145	outdoor LAN: Based on self-reported and organized address information [ArcGIS (ESRI, Redlands, CA)], outdoor LAN data is obtained according to DMSP correspondences. Indoor LAN: Based on self-reports at study enrollment.	Multiple types of breast cancer such as lobular, ductal, and invasive breast cancer were included	35–74	11 years

DCIS, ductal carcinoma in situ; DMSP, Defense Meteorological Satellite Program; ICD-8, International Classification of Diseases, Revision 8; NA, Not stated.

**Table 2:** Summary of main characteristics of the 13 case–control/case-referent studies examining association between exposure to LAN and risk of breast cancer.

Author (year)	Setting	Period	Analysis sample	LAN assessment	Cases	Controls	Age at baseline
Davis S. et al. (2001) (43)	USA	1992–1995	Cases:763 Controls:741	Indoor night light levels were used based on self-reports, and the bedroom ambient light was divided into six levels.	Women diagnosed with breast cancer	female random-digit dialing and were frequency matched according to 5-year age groups	20–74
Fritschi L. et al. (2013) (51)	Australian	2009–2011	Cases:1202 Controls:1785	Based on the self-reported (The Horne-Ostberg) questionnaire of circadian rhythm-related factors, nocturnal light exposure levels were assigned as low, medium, and high according to the algorithm.	Women aged between 18 and 80 years who had a first incident invasive breast cancer	women from the WA electoral roll, and frequency age-matched them to the expected distribution of cases	18–80
Garcia et al. (2018) (37)	Spain	2008–2013	Cases:1599 Controls:1875	Indoor LAN: A questionnaire based on self-reported MCC-Spain was used to reflect the level of indoor night lighting outdoor LAN: LAN exposure based on the geocoded residence with the longest duration for each participant (ISS data in 2012 or 2013)	Cancer cases diagnosed with breast cancer by histology	Cases with no history of cancer and located in the same service area were randomly selected from primary healthcare centers and matched with cases based on gender, 5-year age groups, and study region for frequency matching.	20–85
Keshet et al. (2015) (33)	Israel	2010–2014	Cases:93 Controls:185	Measurement of indoor and outdoor light levels based on self-report questionnaires.	Patients with BC recruited	friends of BC patients and women recruited through personal meetings in schools	29–91
Kloog et al. (2010) (35)	Israel	2006–2008	Cases:794 Controls:885	Self-reported LAN exposure from the sources of outside and household.	Diagnosed with BC	NA	Case: 50.89 (SD = 13.69) Controls: 60.86 (SD = 13.51)
								Li et al. (2010) (40)	USA	1994–1997	Cases:363 Controls:356	Exposure to domestic LAN (Light at Night) in the 10 years before diagnosis or identification (for control group)	ICD-O codes 174.0–174.9	The controls from New Haven County were randomly selected from those who underwent breast-related surgery but who were histologically confirmed with either normal tissue or benign breast diseases without atypia.	30–80
O’Leary et al. (2006) (49)	USA	1996–1997	Cases:487 Controls:509	Self-reported frequency of turning on lights during sleep.	*In situ* or invasive BC	LIBCSP controls were also Long Island residents, frequency matched to the expected age distribution of cases by 5-year age groups.	<75
Ritonja et al. (2020) (48)	Canada	2005–2009	Cases:844 Controls:905	Data from DMSP (2010) and DNB (2012) were utilized, considering participants’ residential histories from 5 to 20 years prior to their enrollment in the study.	Incident *in situ* or invasive breast cancer	Controls were women with either normal mammogram results or a diagnosis of benign breast disease, recruited from the Screening Mammography Program of BC from the same geographic area, frequency matched to cases in 5-year age groups.	40–80
Yang et al. (2019) (42)	China	2013–2016	Cases:401 Controls:401	The cases reported their exposure to nighttime LAN (artificial light at night) during the 5 years before being diagnosed with breast cancer. On the other hand, the controls reported their most recent 5-year bedtime LAN exposure.	Invasive BC	We matched one population control for each case by sex, year of birth (within 1 year), and region of residence (according to the patient’s house number) during the year when the case was diagnosed with cancer.	18–74
Bauer S. E. et al. (2013) (32)	USA	2000–2017	Cases:34053 Controls:14588	The average level of DMSP LAN (artificial light at night) from 1992 to the year of diagnosis was calculated based on the participants’ residential location at the time of diagnosis.	ICD-O-3 C500:509 (2000–07; malignant tumors)	The control group consisted of lung cancer patients obtained from the Georgia Comprehensive Cancer Registry (GCCR)	15–50
Sonia et al. (2020) (44)	Australian	2009–2011	Cases:1205 Controls:1789	Classification of nighttime light exposure based on self-reported questionnaire.	Cases with invasive breast cancer were identified and recruited via the Western Australia Cancer Registry	Controls were randomly selected from the Western Australian electoral roll and frequency age matched to the expected distribution of cases.	18–80
Song et al. (2023) (34)	China	2011–2012	Cases:464 Controls:464	Classification of nighttime light exposure based on self-reported questionnaire.	newly diagnosed breast cancer patients in the Department of Breast Surgery, Cancer Hospital, Chinese Academy of Medical Sciences	histologically confirmed benign breast disease patients collected from the same department during the same period.	44.89(SD = 10.95)
Atalya et al. (2017) (38)	Israel	2010–2014	Cases:110 Controls:142	Classification of nighttime light exposure based on self-reported questionnaire.	Cases were BC patients attending the Comprehensive Cancer Center in Soroka Medical Center (SMC), Beer-Sheva and the Baruch Padeh, Poria Medical Center (PMC) in Tiberius	healthy control subjects with no documented cancer were friends of BC patients and women enrolled through personal meetings in schools.	36–79

DCIS, ductal carcinoma in situ; DMSP, Defense Meteorological Satellite Program; ICD-8, International Classification of Diseases, Revision 8; NA Not stated.

### 3.3 Quality assessment

**Figure 2:**
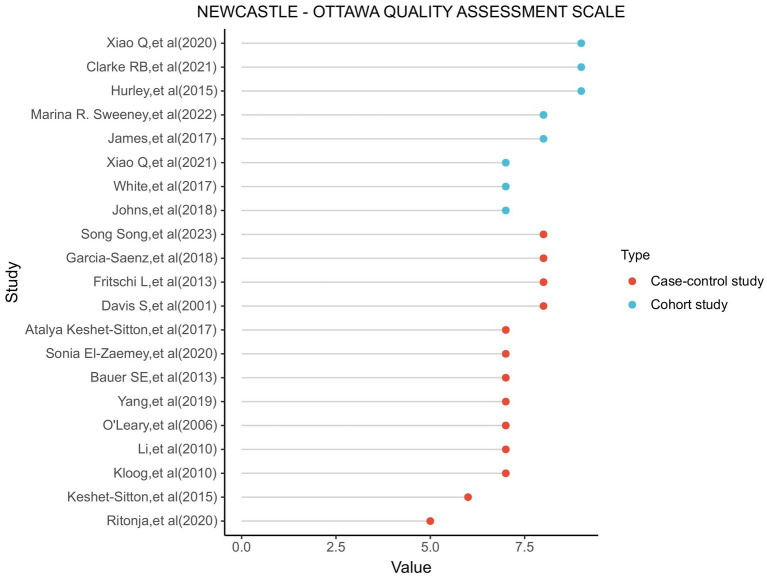
Utilizing the NOS (Newcastle-Ottawa scale) to assess the outcomes of each incorporated study, we evaluate the graphical representation.

In the Newcastle-Ottawa Scale (NOS) assessments conducted on the various studies, the average score for the included research was 7.43 (ranging from 5 to 9). A score of 7 is considered as the cut-off point for high-quality research. Studies with a score of 7 or above are regarded as of higher quality. The scores of the included studies are illustrated in Figure 2.

### 3.4 Light at night exposure and breast cancer risk

**Figure 3:**
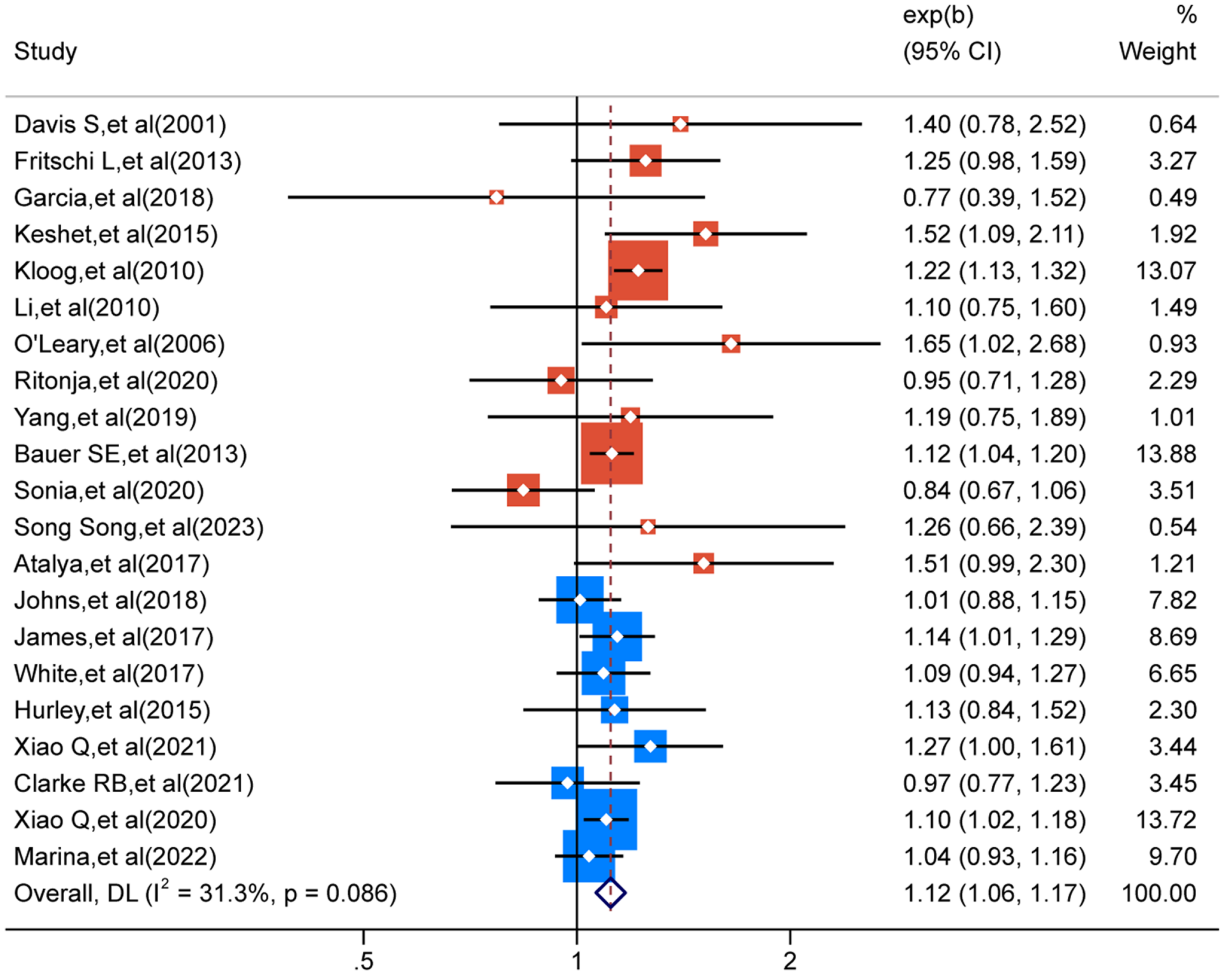
The heterogeneity analysis results for the 21 outcomes concerning nocturnal light exposure.

A comprehensive analysis of 21 studies (32–52) revealed a noteworthy correlation between exposure to LAN and a higher susceptibility to breast cancer (RR = 1.12; 95% CI: 1.06–1.17; I^2^ = 31.3%, *p* < 0.001; Figure 3). Heterogeneity analysis indicated a slight level of heterogeneity in our study, whereas sensitivity analysis did not uncover any individual study findings that overturned the overall results, thus providing evidence for the reliability of our analytical outcomes (Supplementary Figure A).

### 3.5 Subgroup analysis

We conducted subgroup analyses on the 21 included studies to explore the impact of various factors on the incidence of breast cancer (32–52). Based on existing evidence, we categorized these factors into confounding factors (factors that are associated with LAN exposure and breast cancer incidence but are not mediators of the relationship) and effect-modifying factors (those that modify the relationship under different LAN conditions, breast cancer subtypes, or study types). Specifically, confounding factors included geography, menopausal status, BMI, and ethnicity. Effect-modifying factors encompassed study type, LAN (light at night) type, estrogen and progesterone receptor status, and lifestyle. When analyzed as independent confounding factors, no heterogeneity (I^2^ = 0%) was observed for continents, white race, and postmenopausal individuals, which was considered the main factor contributing to the slight heterogeneity observed in the overall analysis.

Regarding confounding factors, in our subgroup analyses that focused on different continents, we observed a clear disparity in breast cancer incidence rates associated with exposure to the LAN. Specifically, the breast cancer incidence rate in Asia (RR = 1.24; 95% CI: 1.15–1.34; I^2^ = 0.0%, *p* < 0.001) was significantly higher than that in North America (RR = 1.11; 95% CI: 1.06–1.15; I^2^ = 0.0%, *p* < 0.001). However, the breast cancer incidence rate in Europe (RR = 0.99; 95% CI: 0.89–1.11; I^2^ = 0.0%, *p* = 0.898), despite being analyzed, did not exhibit any statistically significant difference. When considering racial differences, the incidence of breast cancer was higher in the white population (RR = 1.12; 95% CI: 1.05–1.20; I^2^ = 0.0%, *p* < 0.001), with no statistically significant difference observed in the Black population (RR = 1.15; 95% CI: 0.98–1.35; I^2^ = 4.8%, *p* = 0.080). Additionally, subgroup analysis for menopausal status did not yield statistically significant differences between the premenopausal (RR = 1.09; 95% CI: 0.99–1.19; I^2^ = 17.6%, *p* = 0.083) and postmenopausal groups (RR = 1.03; 95% CI: 0.97–1.09; I^2^ = 0.0%, *p* = 0.352).

In terms of effect-modifying factors, similar to the meta-analysis conducted in 2021 (57), we conducted separate analyses to assess the effects of LAN exposure on breast cancer risk based on estrogen receptor status, types of study, and types of LAN exposure. The analysis of different types of LAN exposure showed that outdoor LAN exposure (RR = 1.07; 95% CI: 1.02–1.13; I^2^ = 30.9%, *p* = 0.004) had a slightly higher promoting effect on breast cancer incidence than indoor LAN exposure (RR = 1.02; 95% CI: 0.92–1.13; I^2^ = 65.6%, *p* = 0.675). In the analysis of estrogen receptor status, LAN exposure exhibited a slightly stronger impact on breast cancer risk for cases with estrogen receptor positivity (RR = 1.10; 95% CI: 1.03–1.18; I^2^ = 17.0%, *p* = 0.005) compared to cases with estrogen receptor negativity (RR = 1.07; 95% CI: 0.94–1.21; I^2^ = 0.0%, *p* = 0.316). Subgroup analyses for different study types revealed that LAN exposure had a higher promoting effect on individual breast cancer incidence in case–control studies (RR = 1.16; 95% CI: 1.06–1.27; I^2^ = 40.4%, *p* = 0.001) compared to cohort studies (RR = 1.08; 95% CI: 1.04–1.14; I^2^ = 0.0%, *p* < 0.001).We also conducted analyses on various lifestyle factors summarized in the literature. When considering TV on while sleeping as an effect-modifying factor, the impact of LAN exposure on breast cancer did not show statistical significance (RR = 1.06; 95% CI: 0.97–1.16; I^2^ = 0.0%, *p* = 0.168). This observation holds true for “Turn on the light when you wake up” (RR = 1.09; 95% CI: 0.87–1.37; I^2^ = 47.5%, *p* = 0.449) and “Bedroom shutters (open)” (RR = 1.07; 95% CI: 0.84–1.37; I^2^ = 64.4%, *p* = 0.589). The complete dataset can be found in Table 3. The results of the Z-tests and the forest plot for the subgroups can be viewed in Supplementary Figure C.

**Table 3:** Subgroup analysis of the risk of breast cancer in patients with exposure to LAN (light at night).

Subgroups	Included studies	OR (95% CI)	Heterogeneity			Z test	
			I^2^(%)	Cochran’s Q	Value of *p*	Model^1^	Model^2^
Study type						*p = 0.05*	*p = 0.23*
Cohort studies	8	1.08(1.04,1.14)	0.0	5.11	0.647		
Case–control studies	13	1.16(1.06,1.27)	40.4	20.12	0.065		
LAN type						*p = 0.95*	*p = 0.30*
Indoor LAN	14	1.02(0.92,1.13)	65.6	37.81	0.000		
outdoor LAN	14	1.07(1.02,1.13)	30.9	18.82	0.129		
Continents						*p < 0.01*	*P < 0.01*
North America	11	1.11(1.06,1.15)	0.0	7.18	0.709		
Europe	3	0.99(0.89,1.11)	0.0	0.64	0.725		
Asia	5	1.24(1.15,1.34)	0.0	2.52	0.642		
Estrogen receptor status						*p = 0.63*	*p = 0.64*
ER+	9	1.10(1.03,1.18)	17.0	9.63	0.292		
ER–	9	1.07(0.94,1.21)	0.0	3.73	0.881		
Menopausal status						*p = 0.36*	*P = 0.36*
Pre-menopausal	9	1.09(0.99,1.19)	17.6	9.71	0.286		
Post-menopausal	10	1.03(0.97,1.09)	0.0	7.54	0.581		
Living habit						*p = 0.87*	*p = 0.99*
TV on while sleeping	4	1.06(0.97,1.16)	0.0	2.08	0.556		
Turn on the light when you wake up	3	1.09(0.87,1.37)	47.5	3.81	0.149		
Bedroom shutters (open)	5	1.07(0.84,1.37)	64.4	11.23	0.024		
BMI						*P = 0.67*	*p = 0.51*
BMI > 25	3	0.98(0.79,1.21)	65.3	5.76	0.056		
BMI < 25	3	1.08(0.90,1.29)	74.6	7.88	0.019		
Progesterone receptor status						*P = 0.87*	*p = 0.83*
PR+	4	0.96(0.84,1.10)	22.2	3.86	0.277		
PR−	4	0.97(0.81,1.18)	0.0	1.54	0.673		
Racial stratification						*p = 0.79*	*p = 0.76*
White	3	1.12(1.05,1.20)	0.0	0.55	0.761		
Black	3	1.15(0.98,1.35)	4.8	2.10	0.350		

ER+, Estrogen receptor-positive; ER−, Estrogen receptor-negative; BMI, Body Mass Index; PR+, Progesterone positive receptor status; PR−, Progesterone negative receptor status; model^1^, Fixed-effect model; model^2^, Random-effect model.

### 3.6 Publication bias

**Figure 4:**
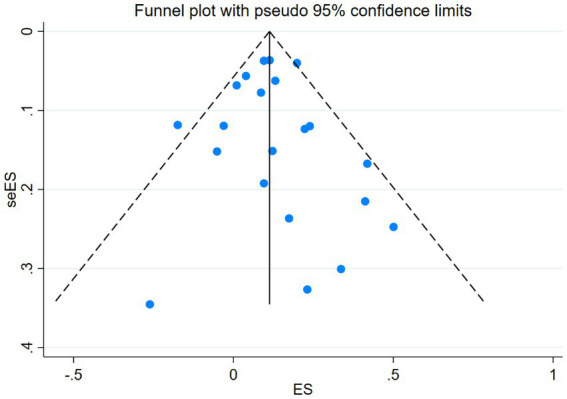
The bias testing results for nocturnal light exposure among the 21 outcome measures.

Visual inspection of the funnel plot did not reveal any significant publication bias in the occurrence of breast cancer. This observation holds true for the overall analysis funnel plot (Figure 4), which is consistent with the results of our Egger’s regression test (*p* = 0.746). Similar findings of bias were observed across various subgroups (Supplementary Figure B).

## 4 Discussion

### 4.1 Main findings

Our meta-analysis included a total of 21 observational studies. The collective data analysis unveiled a significant correlation between exposure to LAN and an escalated susceptibility to breast cancer. Regarding the confounding factors, Jack Sariego first proposed the relationship between geographic correlation and breast cancer (69). This relationship is typically associated with fertility rates and taxation (70). High-quality lifestyles or habits in developed regions are effective in curtailing the incidence of breast cancer (71). We observed that LAN exposure in Asian countries showed the highest increase in breast cancer risk, followed by North America, while in Europe, the breast cancer risk outcome did not reach statistical significance. At the level of menopausal status, many studies have observed a protective effect in premenopausal women in terms of obesity (72), night shift work (73), and physical activity (74). We found that breast cancer risk outcomes did not reach statistical significance, whether in premenopausal or postmenopausal states. We considered additional factors that are associated with LAN exposure and breast cancer incidence but are not mediators of the relationship, such as BMI and race, which could potentially introduce significant bias to the risk outcomes. Multicenter studies have indicated a clear association between higher BMI and breast cancer in women (75), while in terms of race, the annual statistical graphs from the SEER Cancer Statistics Review show consistently higher breast cancer incidence rates in the white population than in the Black population (76). Subgroup analysis for LAN exposure revealed that white individuals have a higher breast cancer risk. Existing evidence supports the notion that cultural dynamics, as well as differences in sociodemographic and behavioral characteristics among different population subgroups, modulate the expression of biological diseases, possibly contributing to the higher breast cancer risk in the white population (14). In terms of modifying factors, previous observational studies have consistently shown a significant increase in breast cancer risk associated with outdoor LAN exposure, particularly among estrogen receptor-positive cases. Our findings align with two prior meta-analyses (57, 60). In a previous meta-analysis, Ka Yan Lai et al. highlighted the impact of indoor LAN exposure in their discussion (60). Lifestyle factors such as window orientation, the use of blinds, and television watching may all contribute to an elevated risk of breast cancer (77). Due to the limited number of studies available, they did not conduct a subgroup analysis in this regard. We have addressed this gap in the literature. Although the five studies included in our analysis did not demonstrate a statistically significant difference, the influence of confounding factors within the lifestyle domain is substantial. Therefore, future, more rigorous research focusing on LAN exposure should be conducted.

### 4.2 Comparison with previous studies

To date, a total of 8 related meta-analyses have been identified (57, 59–62, 64, 78). Regarding the types of included studies, the predominant ones include: four studies assessing the association between breast cancer risk and indoor and outdoor LAN (57.1%) (59–61, 78), four studies investigating the relationship between breast cancer risk and menopausal status (57.1%) (60, 61, 64, 78), and two studies examining the association between breast cancer risk and estrogen receptor status (28.6%) (60, 78).

Ka Yan Lai et al. (60) published a meta-analysis on LAN exposure in 2021. Their results showed a significant association between ER+ and outdoor LAN exposure and cancer risk. They also observed some correlation in premenopausal women. However, they did not consider the potential influence of the patients’ life habits on the experimental results. Furthermore, although their study included participants from seven countries, they did not explore differences in the effects of LAN exposure on breast cancer between countries, although there is solid evidence that cancer risks vary due to economic differences between countries (79). In our analysis, we supplemented subgroups with continental differences and lifestyle habits. We did not observe significant differences in LAN exposure and breast cancer risk by different menopausal states. Therefore, our study provides more subgroup analysis and new evidence in this field, which has certain reference value.

Another meta-analysis, conducted by Teresa Urbano et al. (57), encompassed 17 studies. Subgroup analysis was performed based on study type, menopausal status, and estrogen receptor (ER) status. Their findings revealed a noteworthy elevation in the risk of breast cancer attributed to exposure to LAN in cohort studies, premenopausal women, and ER-positive women. In contrast, our study employed a larger sample size (*n* = 21). Notably, our findings demonstrated a more substantial risk association among the case–control study population, contradicting the results reported by Urbano et al. (57).

### 4.3 Interpretation of findings

The existing three hypotheses attempt to explain the connection between LAN exposure and the risk of various cancers: the direct inhibition of melatonin secretion (80), sleep deprivation affecting cell proliferation and cytokine production (81), and the effect of chrono disruption (82, 83). The function of melatonin was described in detail in 2018 (84). Melatonin is considered a pleiotropic and multitasking molecule (85). In addition to regulating circadian rhythms, it plays a significant role in anticancer effects. It inhibits tumor cell proliferation and invasion, suppresses DNA damage, and modulates the immune system to aid in the elimination of tumor cells. All these factors support its inhibitory effect on breast cancer (55, 81). Additionally, some observational studies have confirmed the suppression of melatonin secretion due to indoor LAN exposure (86). In addition, we should also acknowledge the potential impacts of sleep deprivation and circadian disruption. The sleep–wake cycle is a natural component of human life (87), including waking up during the day and sleeping overnight. The sleep pattern is controlled by static sleep pressure and the circadian rhythm (88), so as the day progresses, the combined effect of these two factors makes it easier for us to fall asleep, while signals from the circadian rhythm can prevent us from falling asleep. Experimental evidence has shown that sleep deprivation can significantly impair learning, memory, judgment, and concentration (89). In terms of health-related risk factors, according to the Nurse’s Health Study, shift work nurses, especially those who work at least three times a month for 15 years or more, have a significantly increased risk of colorectal cancer (90). The risk of breast cancer in nurses who have worked in shift rotations for over 20 years is 1.79 times higher (91). The detrimental effects of LAN exposure on sleep have been confirmed in the experiments conducted by Yu-Xiang Xu et al. (92).

### 4.4 Implications and limitations

Nonetheless, our meta-analysis has limitations. First, as is typical with observational studies, uncontrolled confounding factors can affect our evaluations. When analyzing lifestyle factors, we did not consider potential influences from factors such as “turn on the light when you wake up” due to insufficient descriptions or sample sizes in observational studies. To control for confounding factors within acceptable limits, we did not assess the potential impact of LAN exposure during night shifts or rotating shift work on breast cancer. The impacts of insufficient sleep duration and sleep quality were not explored, despite prior research showing their significance (93).Other established risk factors for breast cancer, such as noise, air quality, smoking, and alcohol intake, were not adequately examined due to small sample sizes. For instance, observational studies on alcohol accounted for only 4.8% (*n* = 1) (47), and subgroup studies on smoking were similarly limited at 9.5% (*n* = 2) (46, 47). While the influence of BMI on breast cancer has been established (94), we could not analyze its association with LAN exposure due to the limited data available. The use of personal electronic devices, lightning, and magnetic fields represent potential sources of exposure that should be considered. Second, due to inconsistent indicators of outdoor LAN exposure data of various materials, DMSP (47) or DNP (48) was used for LAN dose detection in existing experiments, and no updated outdoor LAN exposure dose data were available after 2021. Therefore, we did not conduct a dose analysis on the correlation between LAN exposure and breast cancer. Additionally, it is inevitable that we included retrospective cohort studies and case–control studies, where patients’ subjective descriptions may introduce potential biases and ultimately lead to recall bias. Therefore, the interpretation of the results should be approached with caution. The last factor is the subjective design error. In many indoor LAN exposure studies based on self-reporting (35–39, 43, 50, 51), statistical analysis is conducted to compare the population with the highest LAN exposure or presence of LAN exposure to those without LAN exposure or with the lowest dose. However, due to different questionnaire designs and varying definitions of high and low doses (for example, Davis et al. (43) categorized indoor LAN exposure into six levels, while Johns et al. (36) used only three levels), the subjectivity present in each study cannot be avoided in our selection. Undeniably, our study still has many strengths. (1) Following the suggestion of Ka Yan Lai et al. (60), we have, for the first time, considered continents and lifestyle habits in the meta-analysis of LAN exposure and breast cancer and obtained favorable results. (2) Our study supports the claim that there is no significant difference in breast cancer risk between pre- and postmenopausal women exposed to the LAN, as we did not observe any statistical significance in our meta-analysis, providing new evidence for this factor. (3) Our study found that individuals with high exposure to the LAN, especially among Asian populations, are more susceptible to breast cancer. Therefore, it may be of great importance for relevant public health agencies to implement necessary protective measures and preventive strategies for specific groups. Therefore, future research should incorporate relevant influencing factors, particularly those related to unhealthy lifestyle choices. Additionally, designing standardized indoor LAN exposure assessments will aid in evaluating factors associated with breast cancer risk.

## 5 Conclusion

Our meta-analysis provides evidence to support the hypothesis that exposure to LAN is associated with an elevated risk of breast cancer incidence, thereby adding valuable insights to the existing body of research. Although we did not observe a statistically significant correlation between lifestyle factors and the association between LAN and breast cancer incidence, our findings indicate a stronger relationship in certain subgroups, such as study populations located in Asia or investigations focusing on outdoor LAN exposure. These results contribute to the identification of important avenues for future research and offer meaningful recommendations.

## Author contributions

ZIL: Conceptualization, Data curation, Formal analysis, Software, Supervision, Validation, Writing – original draft, Writing – review & editing. ZHL: Conceptualization, Funding–acquisition, Writing – review & editing. HC: Data curation, Investigation, Supervision, Writing – review & editing. YL: Writing – review & editing-Methodology/Investigation. NT: Investigation, Supervision, Writing – review & editing. HL: Investigation, Supervision, Writing – review & editing.
