# Supplementary material for: Light at night exposure and risk of breast cancer: a meta-analysis of observational studies
Source: Front Public Health. 2023 Dec 1;11:1276290. doi: 10.3389/fpubh.2023.1276290 (PMC10722424; doi:10.3389/fpubh.2023.1276290)
Supplement: Supplementary file 1 [file Data_Sheet_1.doc]

**Supplementary**

**Table** **1~3: Details of the Literature Search Strategy**

(1) PubMed (July 15, 2023)

| **Search** | **Query** | **Results** |
| --- | --- | --- |
| #1 | **((((((((((((((((light exposure*[Title/Abstract]) OR (radiation exposure[Title/Abstract])) OR (sun exposure[Title/Abstract])) OR (indoor light[Title/Abstract])) OR (outdoor light[Title/Abstract])) OR (light pollution[Title/Abstract])) OR (light[Title/Abstract])) OR (light at night*[Title/Abstract])) OR (light intensity[Title/Abstract])) OR (light related phenomena[Title/Abstract])) OR (illumination*[Title/Abstract])) OR (nightlight[Title/Abstract])) OR (nighttime light[Title/Abstract])) OR (ionising radiation[Title/Abstract])) OR (ultraviolet radiation[Title/Abstract])) OR (cosmic radiation[Title/Abstract])) OR (solar radiation[Title/Abstract])** | 869,746 |
| #2 | **"Breast Neoplasms"[Mesh]** | 342,097 |
| #3 | **(((((((((((Breast Neoplasm*[Title/Abstract]) OR (Breast Tumor*[Title/Abstract])) OR (Breast Cancer*[Title/Abstract])) OR (Mammary Cancer*[Title/Abstract])) OR (Malignant Neoplasm of Breast[Title/Abstract])) OR (Breast Malignant Neoplasm*[Title/Abstract])) OR (Malignant Tumor of Breast[Title/Abstract])) OR (Breast Malignant Tumor*[Title/Abstract])) OR (Cancer of Breast[Title/Abstract])) OR (Human Mammary Carcinoma*[Title/Abstract])) OR (Human Mammary Neoplasm*[Title/Abstract])) OR (Breast Carcinoma*[Title/Abstract])** | 373,737 |
| #4 | **((((((((((((Breast Neoplasm*[Title/Abstract]) OR (Breast Tumor*[Title/Abstract])) OR (Breast Cancer*[Title/Abstract])) OR (Mammary Cancer*[Title/Abstract])) OR (Malignant Neoplasm of Breast[Title/Abstract])) OR (Breast Malignant Neoplasm*[Title/Abstract])) OR (Malignant Tumor of Breast[Title/Abstract])) OR (Breast Malignant Tumor*[Title/Abstract])) OR (Cancer of Breast[Title/Abstract])) OR (Human Mammary Carcinoma*[Title/Abstract])) OR (Human Mammary Neoplasm*[Title/Abstract])) OR (Breast Carcinoma*[Title/Abstract])) OR ("Breast Neoplasms"[Mesh])** | 453,793 |
| #5 | **(risk[Title/Abstract]) OR (risk[MeSH])** | 3,256,291 |
| #6 | **(((risk[Title/Abstract]) OR (risk[MeSH])) AND (((((((((((((Breast Neoplasm*[Title/Abstract]) OR (Breast Tumor*[Title/Abstract])) OR (Breast Cancer*[Title/Abstract])) OR (Mammary Cancer*[Title/Abstract])) OR (Malignant Neoplasm of Breast[Title/Abstract])) OR (Breast Malignant Neoplasm*[Title/Abstract])) OR (Malignant Tumor of Breast[Title/Abstract])) OR (Breast Malignant Tumor*[Title/Abstract])) OR (Cancer of Breast[Title/Abstract])) OR (Human Mammary Carcinoma*[Title/Abstract])) OR (Human Mammary Neoplasm*[Title/Abstract])) OR (Breast Carcinoma*[Title/Abstract])) OR ("Breast Neoplasms"[Mesh]))) AND (((((((((((((((((light exposure*[Title/Abstract]) OR (radiation exposure[Title/Abstract])) OR (sun exposure[Title/Abstract])) OR (indoor light[Title/Abstract])) OR (outdoor light[Title/Abstract])) OR (light pollution[Title/Abstract])) OR (light[Title/Abstract])) OR (light at night*[Title/Abstract])) OR (light intensity[Title/Abstract])) OR (light related phenomena[Title/Abstract])) OR (illumination*[Title/Abstract])) OR (nightlight[Title/Abstract])) OR (nighttime light[Title/Abstract])) OR (ionising radiation[Title/Abstract])) OR (ultraviolet radiation[Title/Abstract])) OR (cosmic radiation[Title/Abstract])) OR (solar radiation[Title/Abstract]))** | 1,502 |

(2) Cochrane Library (July 15, 2023)

| **Search** | **Query** | **Results** |
| --- | --- | --- |
| #1 | (light exposure*):ti,ab,kw OR (radiation exposure):ti,ab,kw OR (sun exposure):ti,ab,kw OR (indoor light):ti,ab,kw OR (outdoor light):ti,ab,kw OR (light pollution):ti,ab,kw OR (light):ti,ab,kw OR (light at night*):ti,ab,kw OR (light intensity):ti,ab,kw OR (light related phenomena):ti,ab,kw OR (illumination*):ti,ab,kw OR (nightlight):ti,ab,kw OR (nighttime light):ti,ab,kw OR (ionising radiation):ti,ab,kw OR (ultraviolet radiation):ti,ab,kw OR (cosmic radiation):ti,ab,kw OR (solar radiation):ti,ab,kw | 32,225 |
| #2 | MeSH descriptor: [Breast Neoplasms] explode all trees | 17,876 |
| #3 | (Breast Neoplasm*):ti,ab,kw OR (Breast Tumor*):ti,ab,kw OR (Breast Cancer*):ti,ab,kw OR (Mammary Cancer*):ti,ab,kw OR (Malignant Neoplasm of Breast):ti,ab,kw OR (Breast Malignant Neoplasm*):ti,ab,kw OR (Malignant Tumor of Breast):ti,ab,kw OR (Breast Malignant Tumor*):ti,ab,kw OR (Cancer of Breast):ti,ab,kw OR (Human Mammary Carcinoma*):ti,ab,kw OR (Human Mammary Neoplasm*):ti,ab,kw OR (Breast Carcinoma*):ti,ab,kw | 45,584 |
| #4 | #2 OR #3 | 45,584 |
| #5 | MeSH descriptor: [Risk] explode all trees OR (Risk):ti,ab,kw | 288,349 |
| #6 | #5 AND #4 AND #1 | 175 |

(3) Embase (July 15, 2023)

| **Search** | **Query** | **Items found** |
| --- | --- | --- |
| #1 | 'light exposure*':ti,ab,kw OR 'radiation exposure':ti,ab,kw OR 'sun exposure':ti,ab,kw OR 'indoor light':ti,ab,kw OR 'outdoor light':ti,ab,kw OR 'light pollution':ti,ab,kw OR light:ti,ab,kw OR 'light at night*':ti,ab,kw OR 'light intensity':ti,ab,kw OR 'light related phenomena':ti,ab,kw OR illumination*:ti,ab,kw OR nightlight:ti,ab,kw OR 'nighttime light':ti,ab,kw OR 'ionising radiation':ti,ab,kw OR 'ultraviolet radiation':ti,ab,kw OR 'cosmic radiation':ti,ab,kw OR 'solar radiation':ti,ab,kw | 965,256 |
| #2 | 'breast tumor'/exp | 673,592 |
| #3 | 'breast neoplasm*':ti,ab,kw OR 'breast tumor*':ti,ab,kw OR 'breast cancer*':ti,ab,kw OR 'mammary cancer*':ti,ab,kw OR 'malignant neoplasm of breast':ti,ab,kw OR 'breast malignant neoplasm*':ti,ab,kw OR 'malignant tumor of breast':ti,ab,kw OR 'breast malignant tumor*':ti,ab,kw OR 'cancer of breast':ti,ab,kw OR 'human mammary carcinoma*':ti,ab,kw OR 'human mammary neoplasm*':ti,ab,kw OR 'breast carcinoma*':ti,ab,kw | 539,936 |
| #4 | #2 OR #3 | 734,316 |
| #5 | 'risk'/exp | 3,065,357 |
| #6 | 'risk':ti,ab,kw | 3,972,787 |
| #7 | #5 OR #6 | 4,860,082 |
| #8 | #1 AND #4 AND #7 | 2,706 |

**Figure A:** The sensitivity analysis results for nocturnal light exposure among the 21 outcome measures.

**
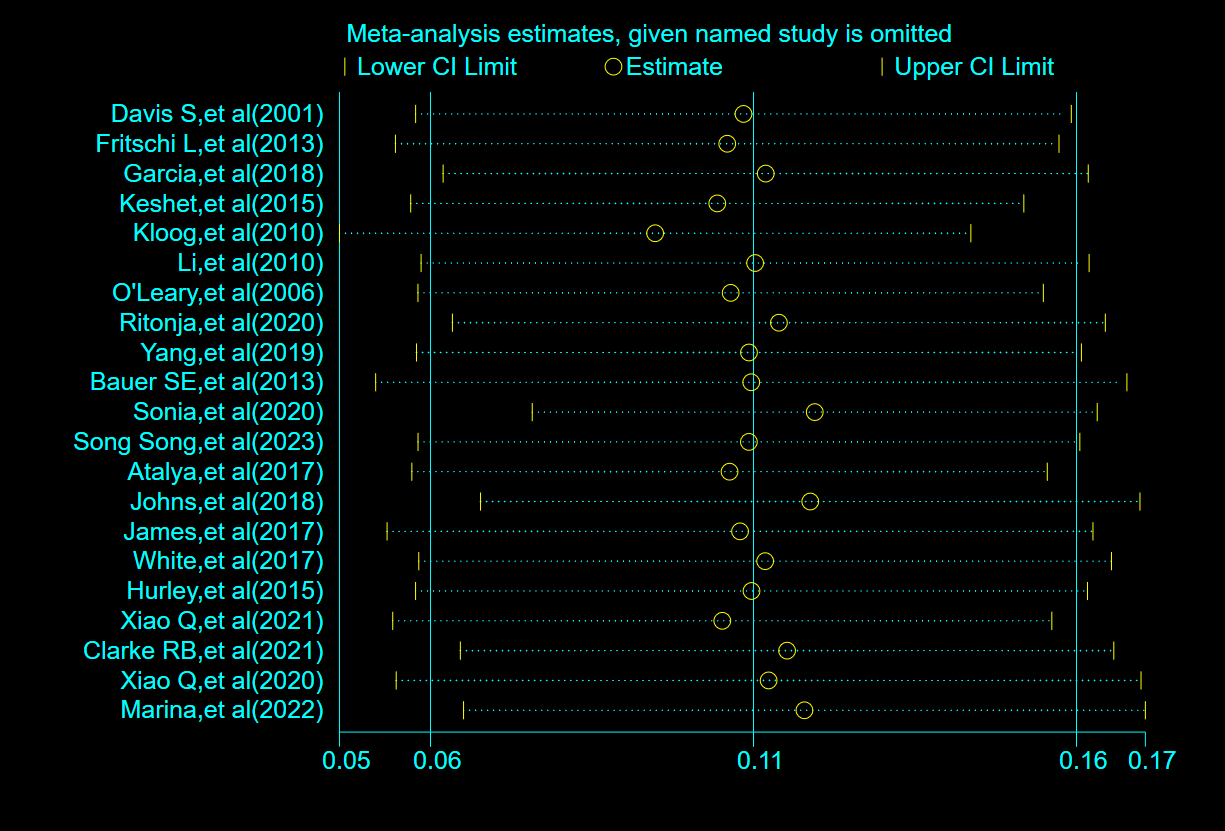
**

**Figure B:** Bias testing results among multiple subgroups of LAN exposure.

| **Study type** | |
| --- | --- |
| Cohort studies | Case-control studies |
| 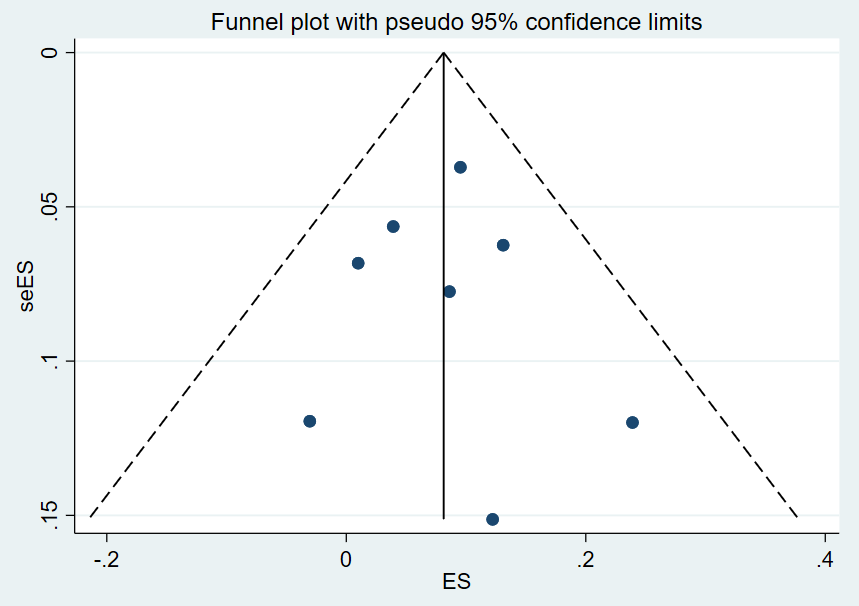 | 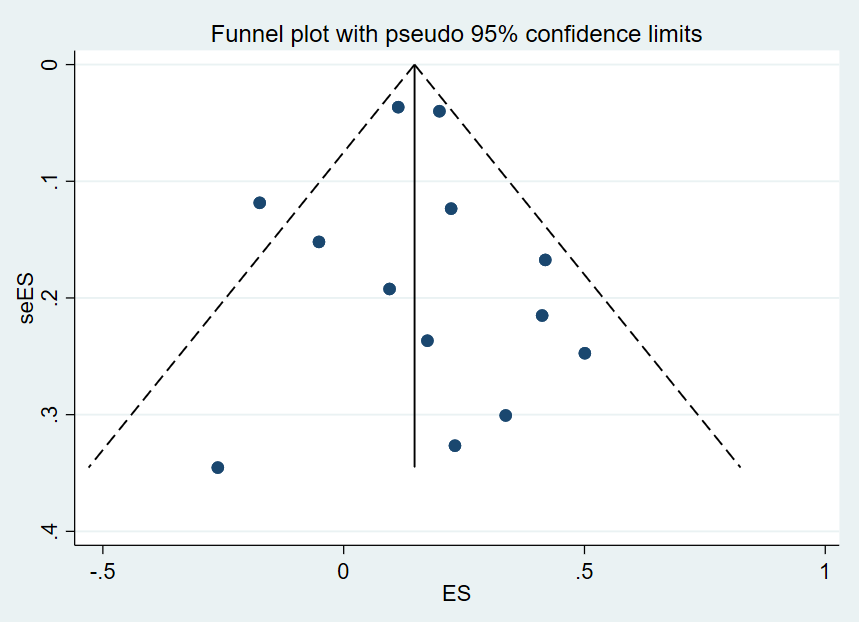 |

| **LAN type** | |
| --- | --- |
| Indoor LAN | outdoor LAN |
| 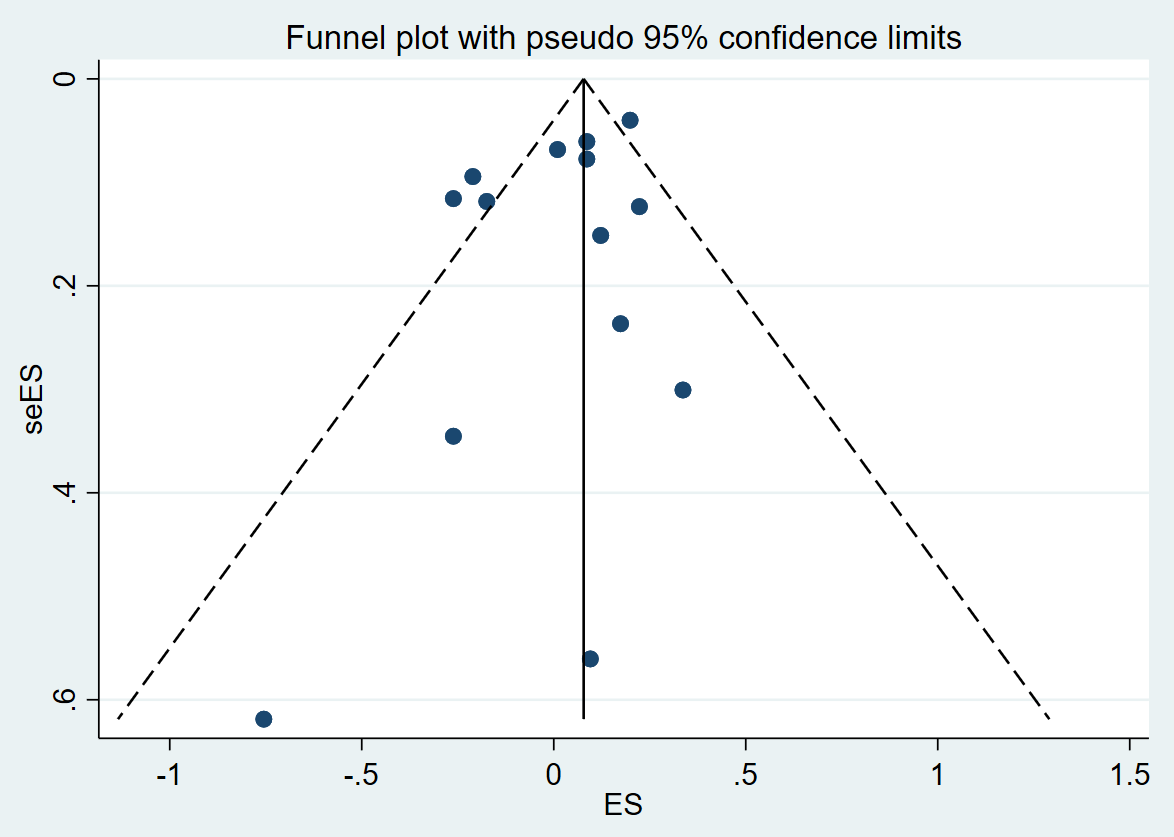 | 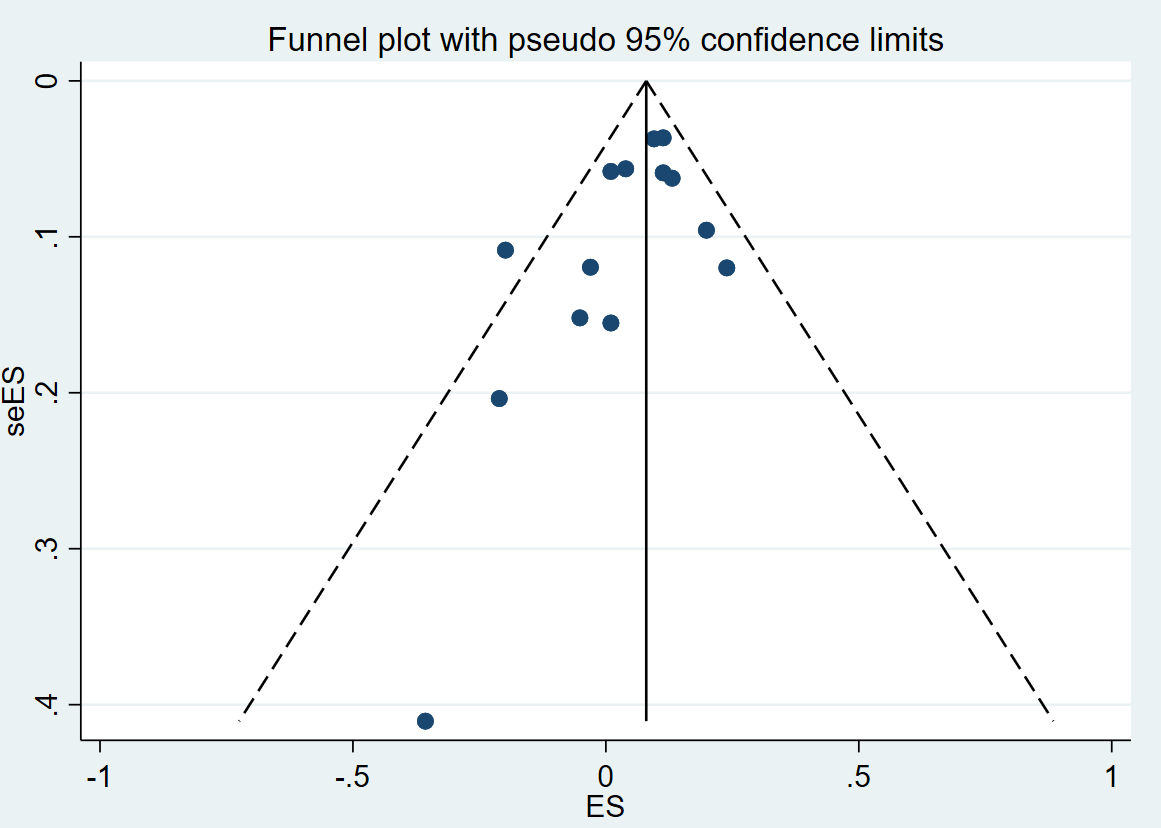 |

| **Estrogen receptor status** | |
| --- | --- |
| ER+ | ER- |
| 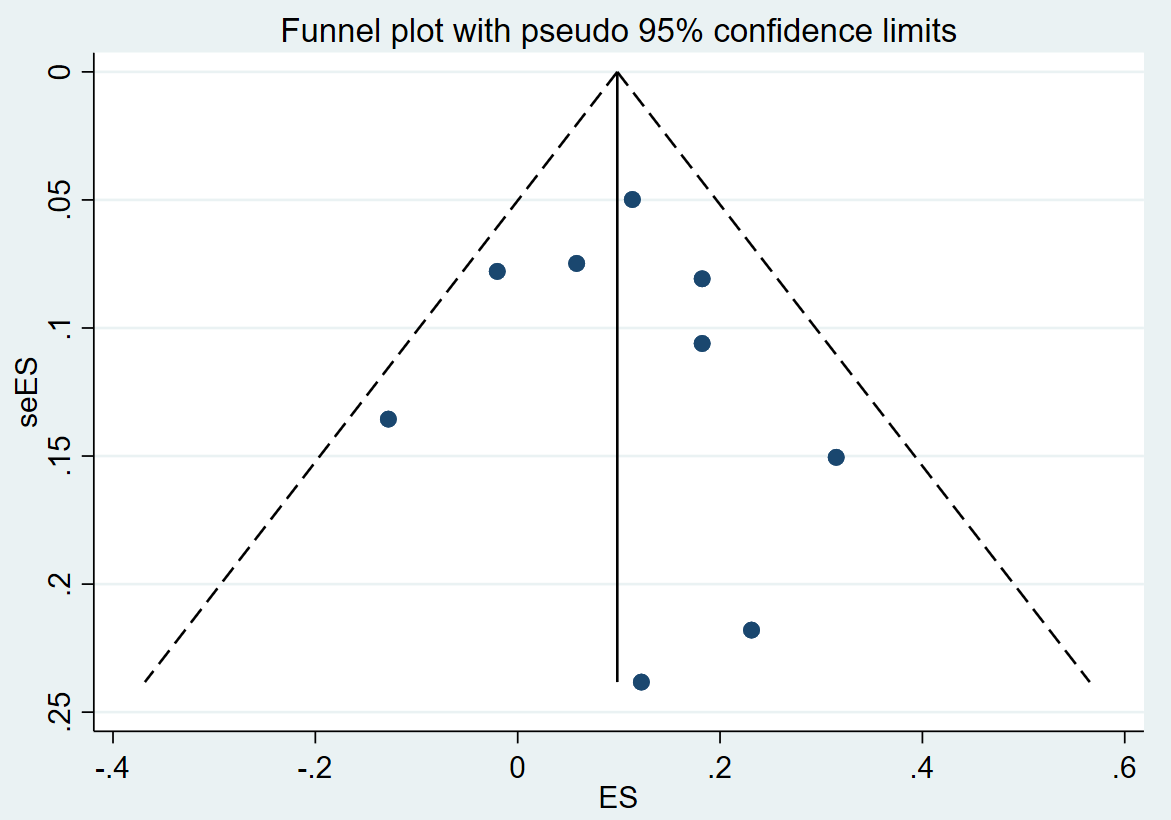 | 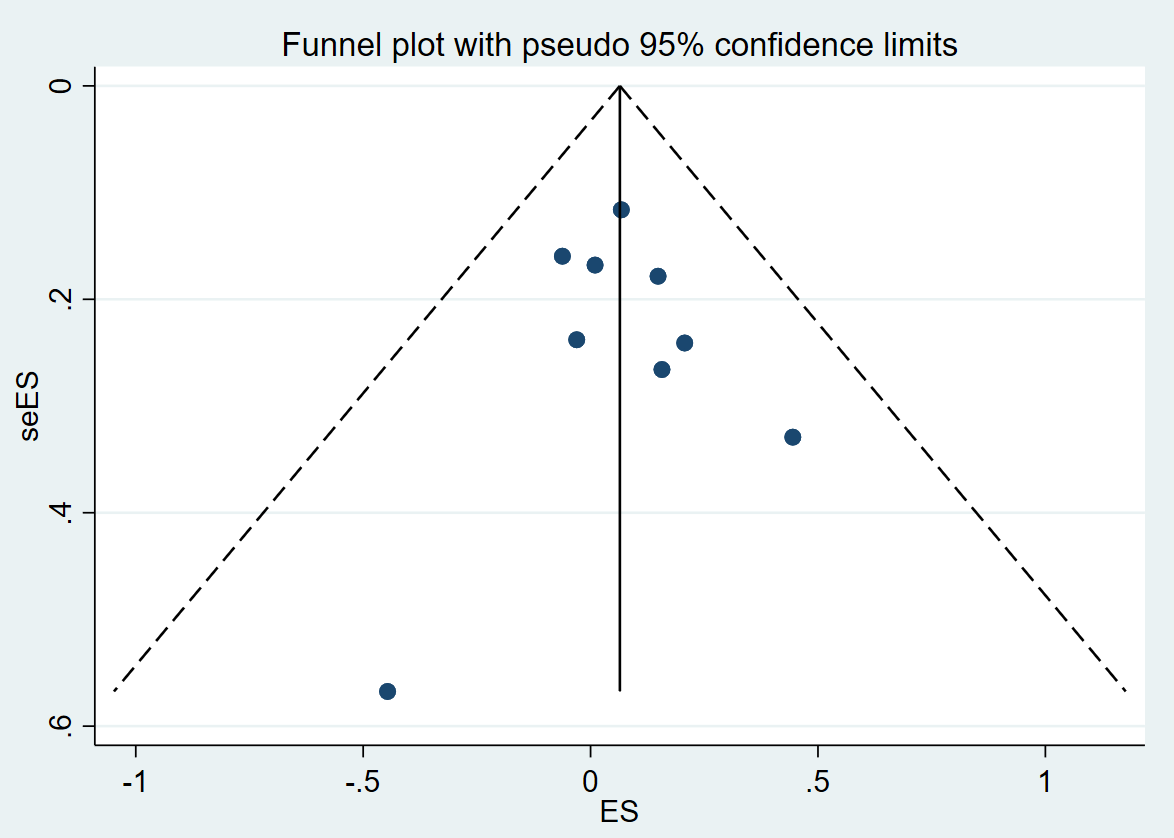 |

| **Menopausal status** | |
| --- | --- |
| Pre-menopausal | Post-menopausal |
| 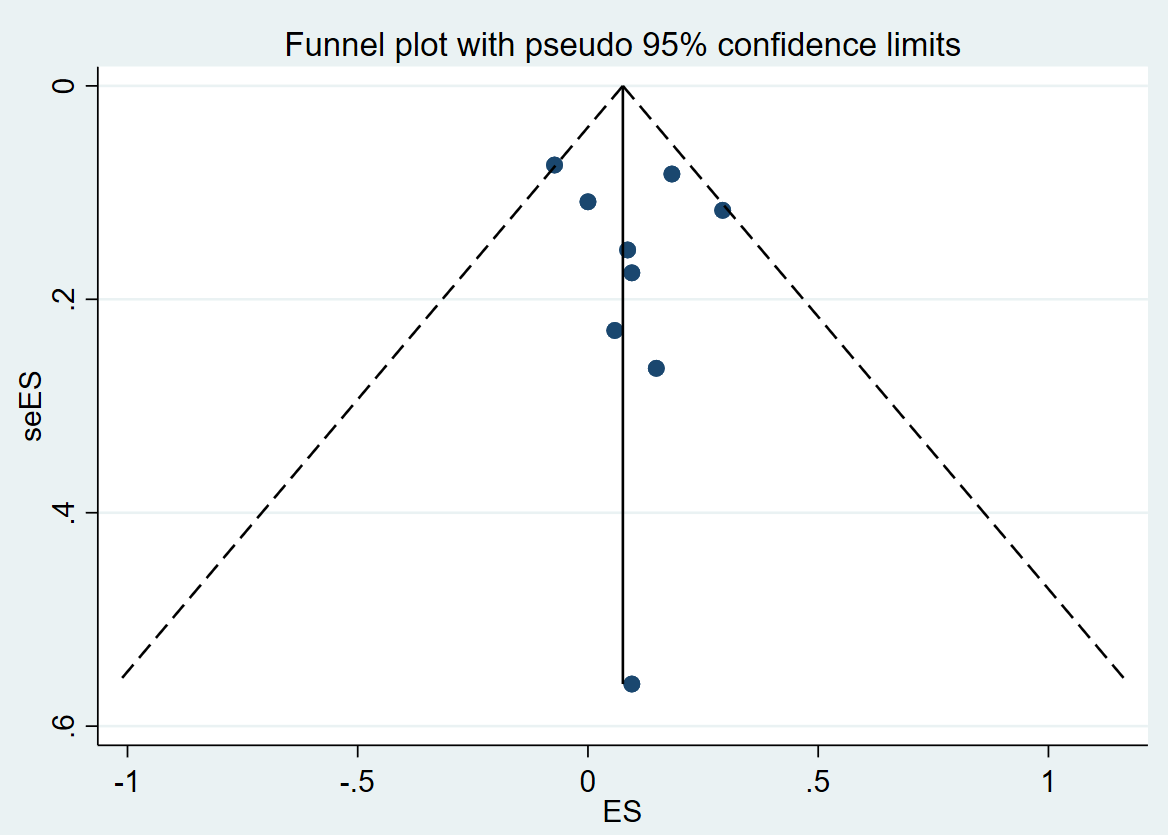 | 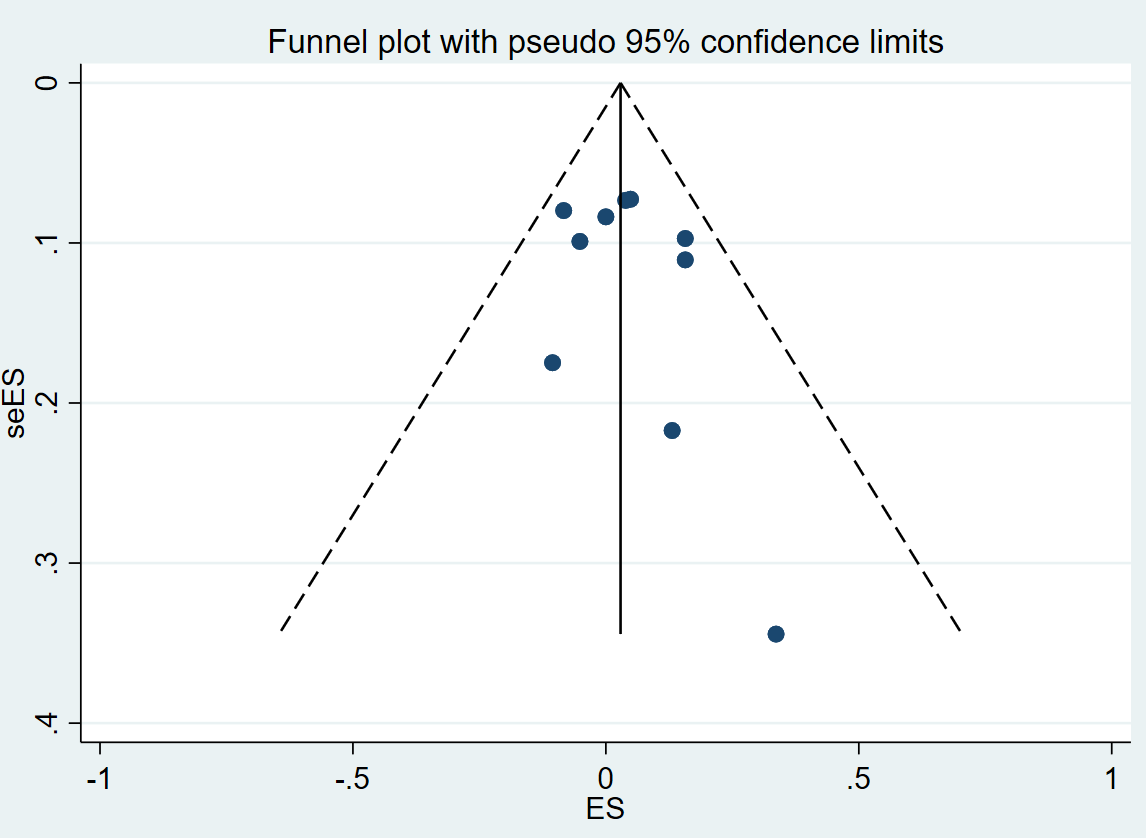 |

| **Continents** | | |
| --- | --- | --- |
| North America | Europe | Asia |
| 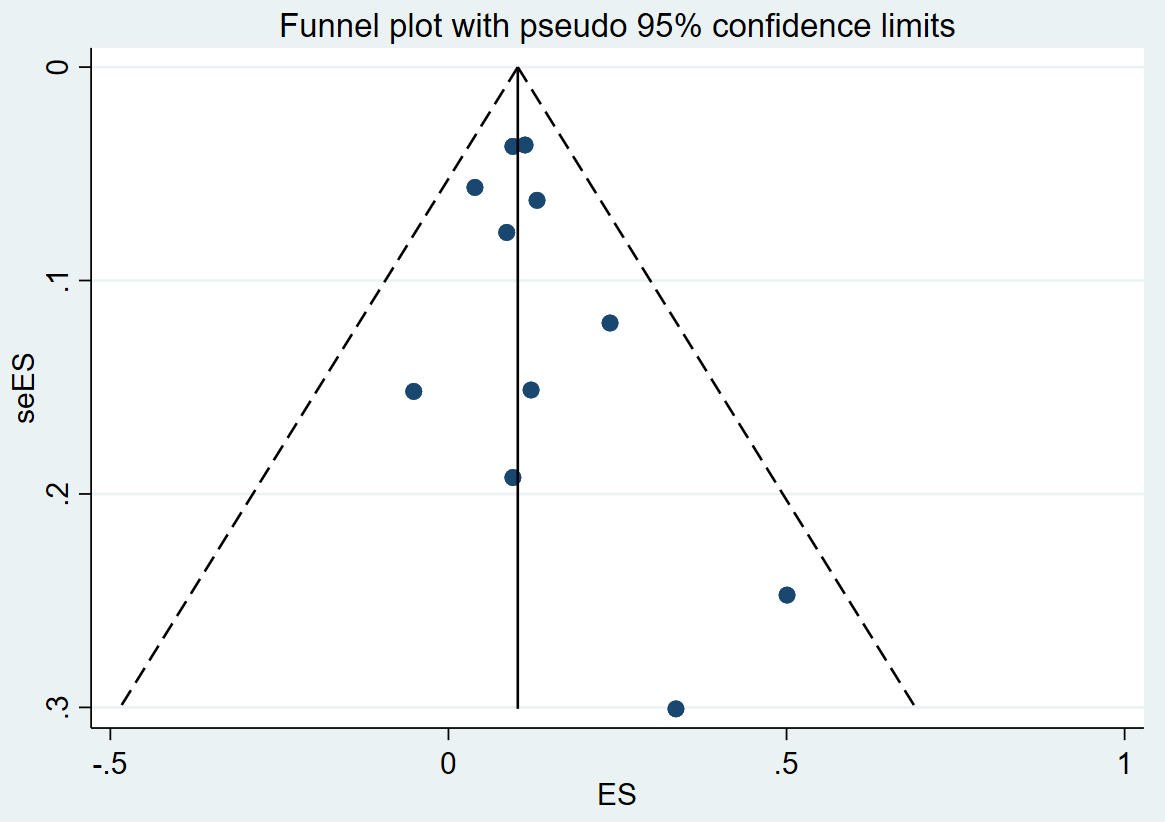 | 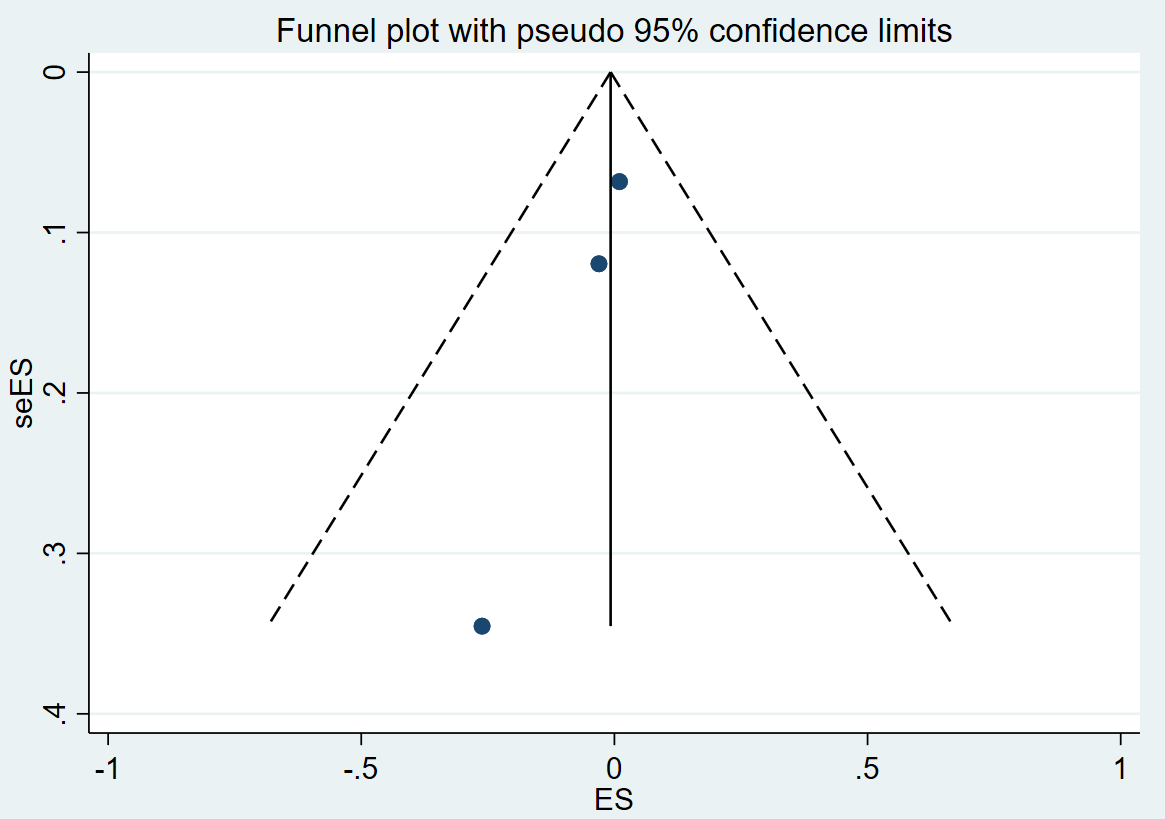 | 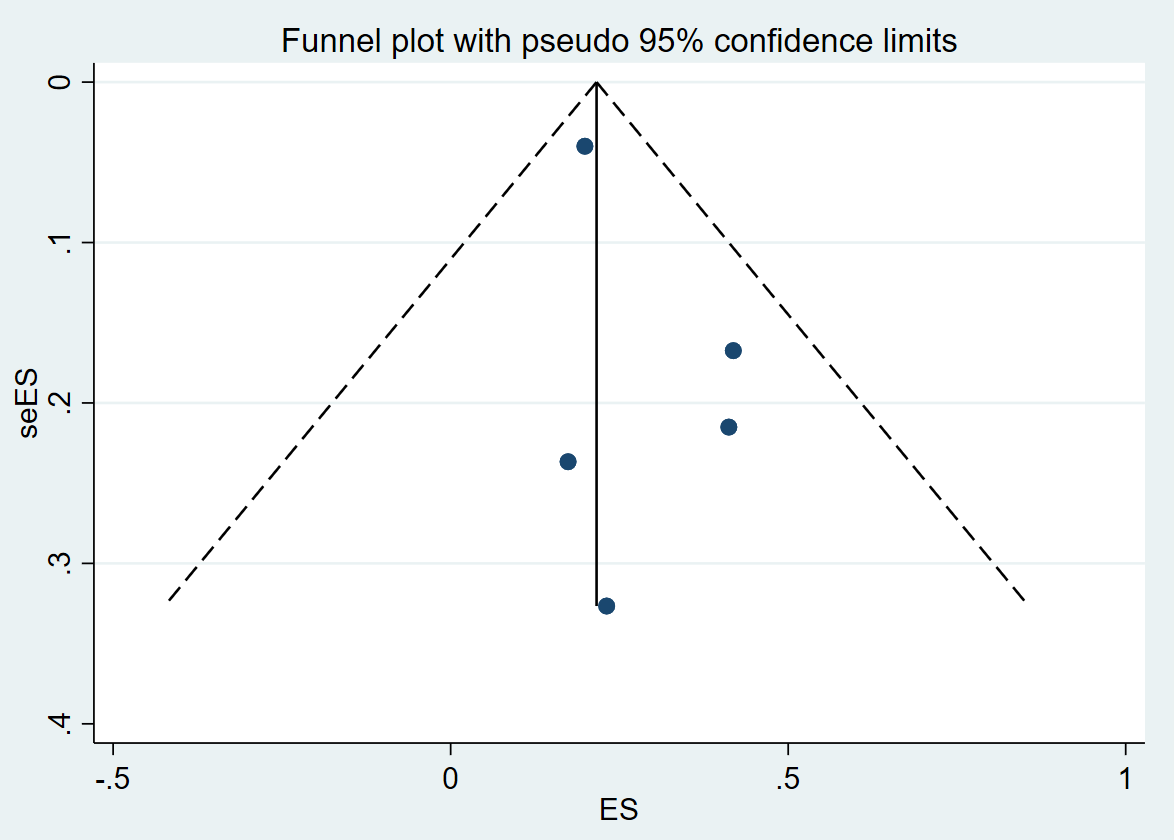 |

| **Living habit** | | |
| --- | --- | --- |
| TV on while sleeping | Turn on the light when you wake up | Bedroom shutters (open) |
| 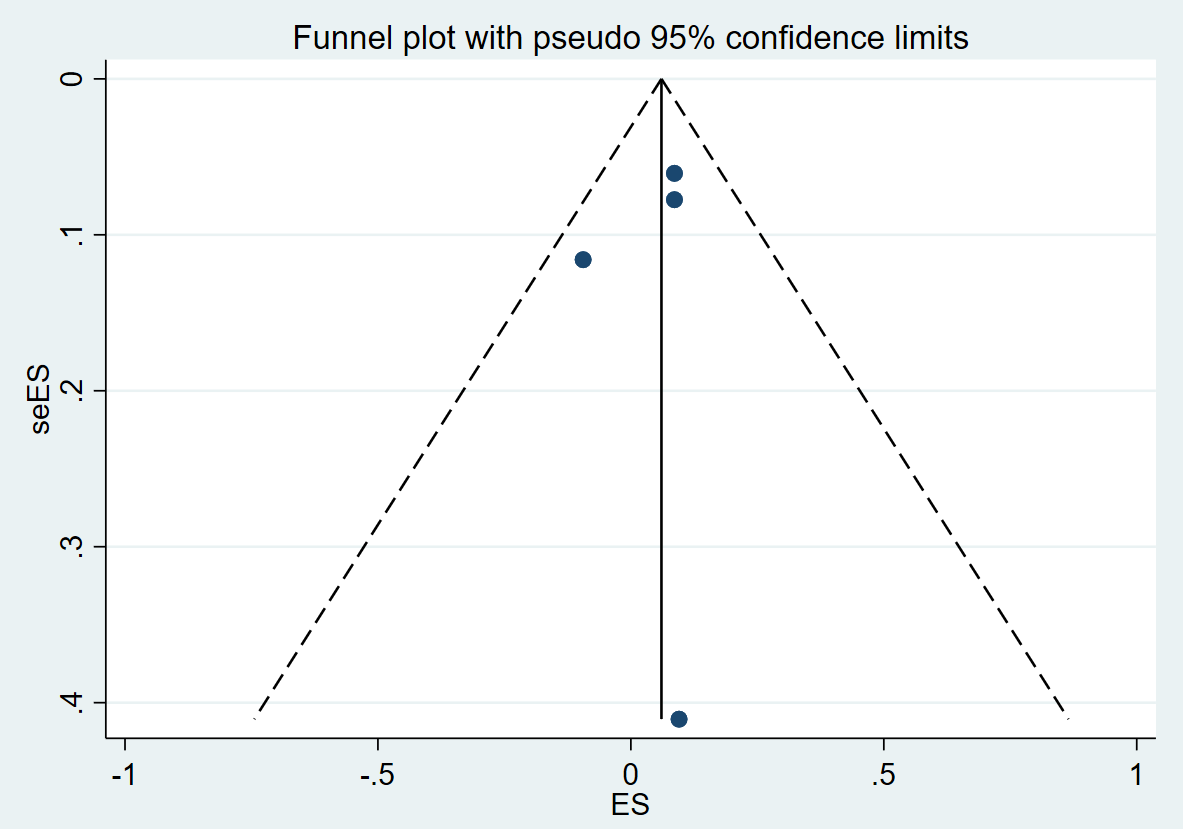 | 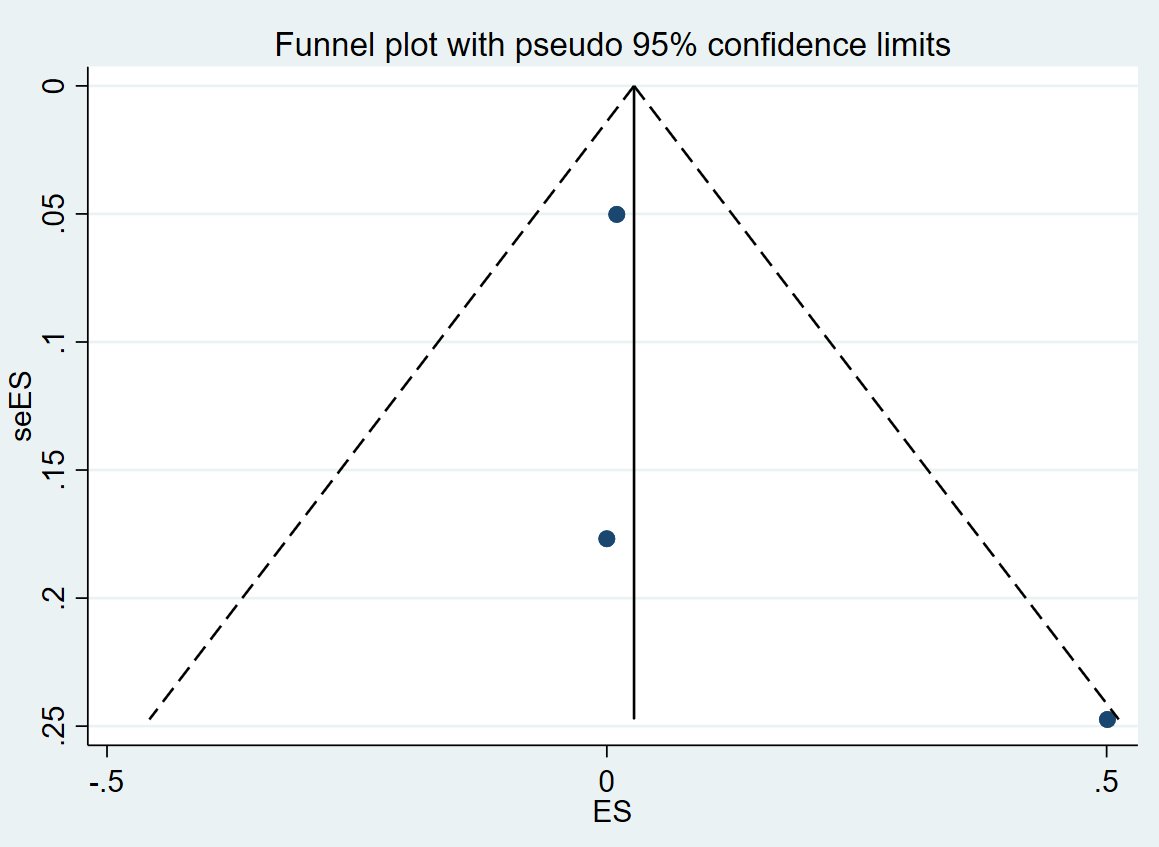 | 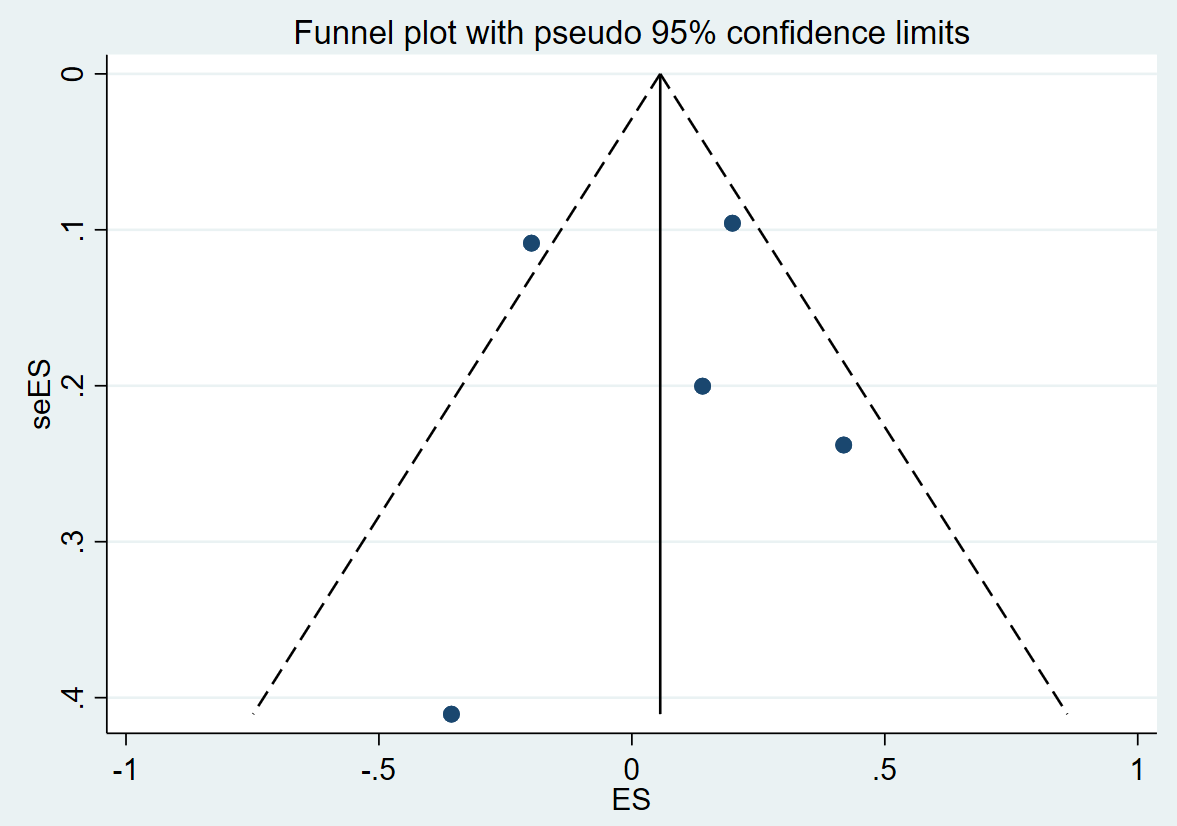 |

| **Progesterone receptor status** | |
| --- | --- |
| PR+ | PR- |
| 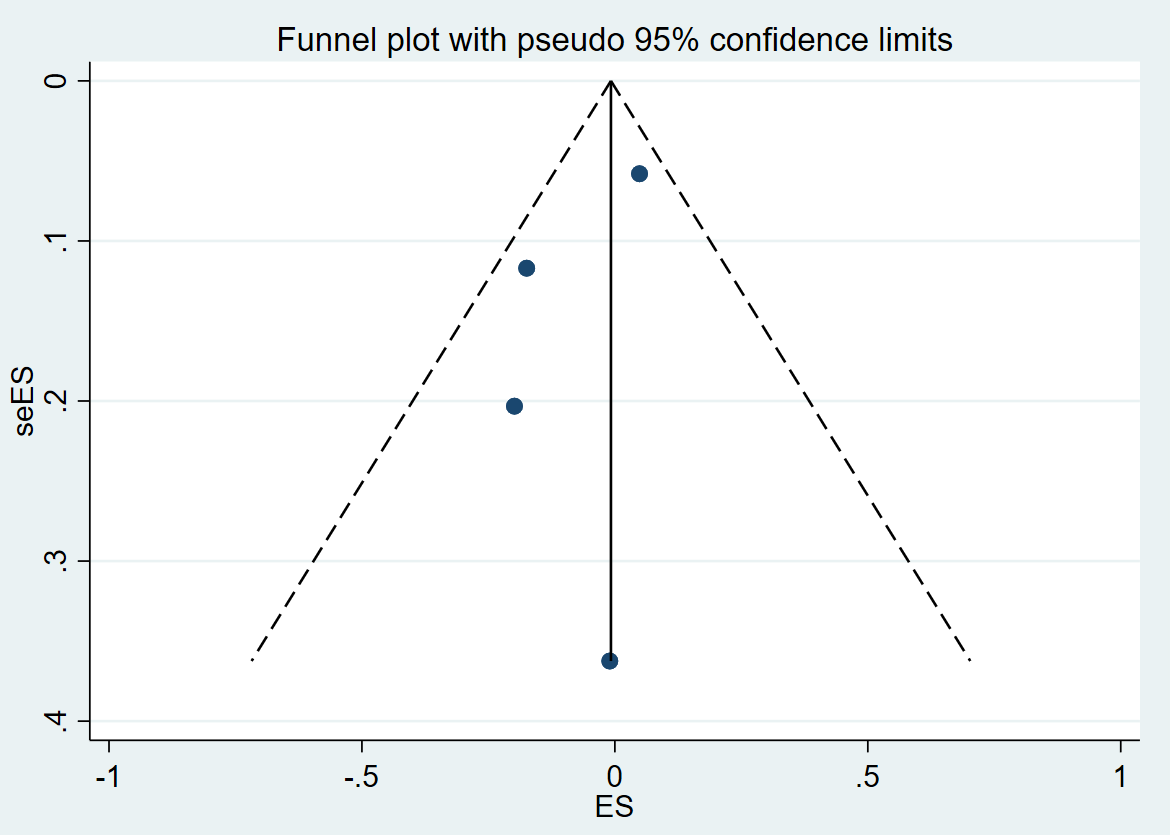 | 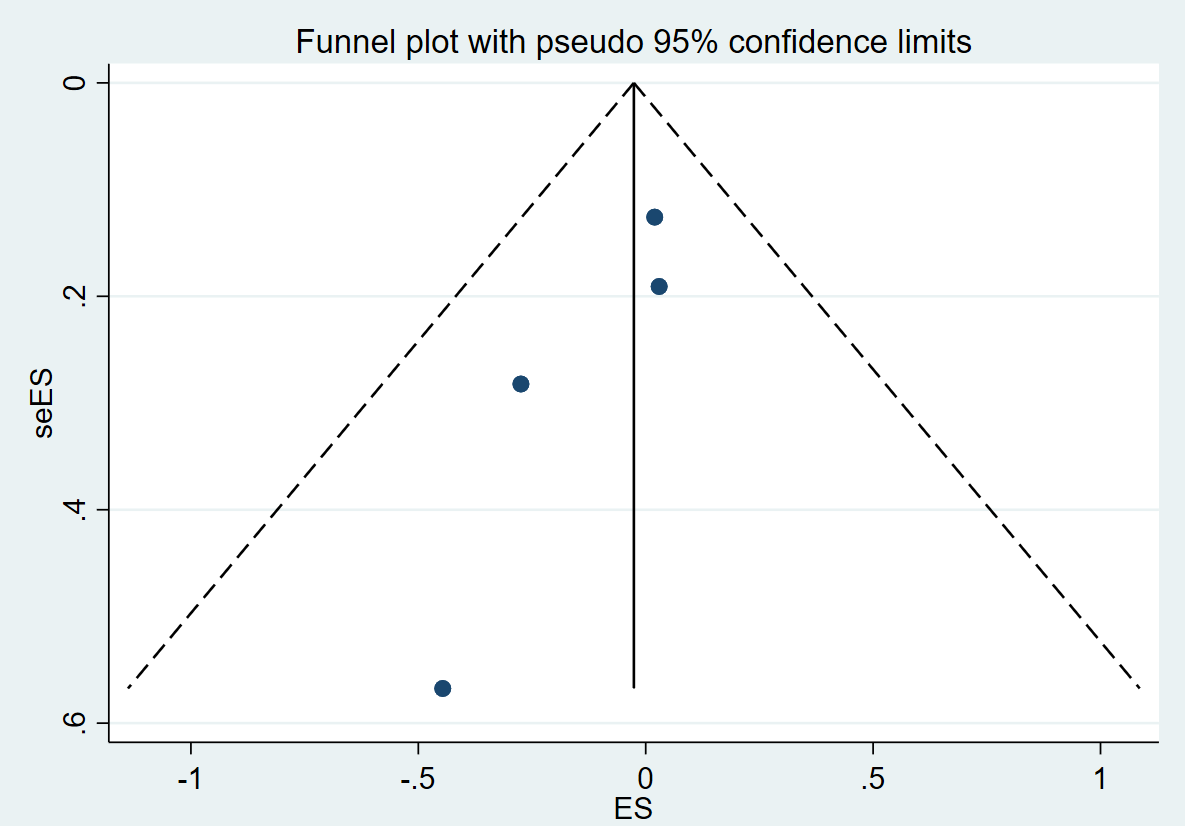 |

| **BMI** | |
| --- | --- |
| BMI>25 | BMI<25 |
| 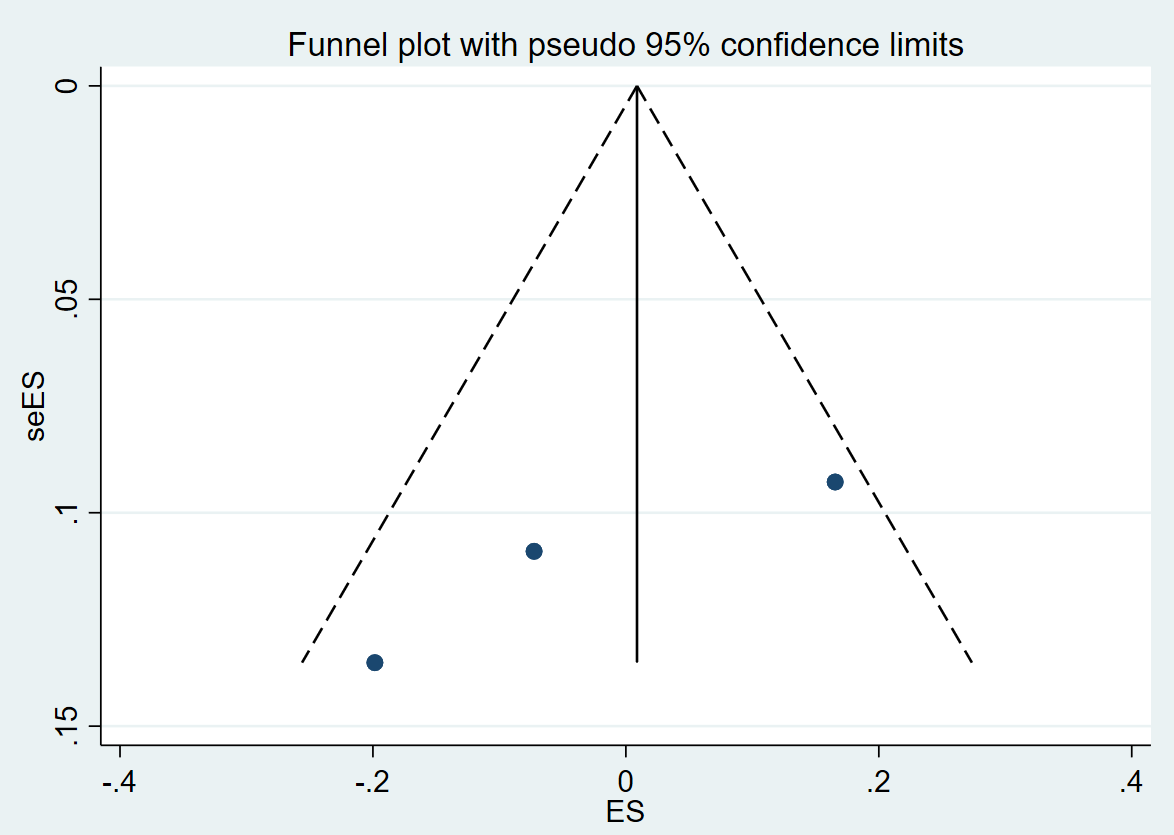 | 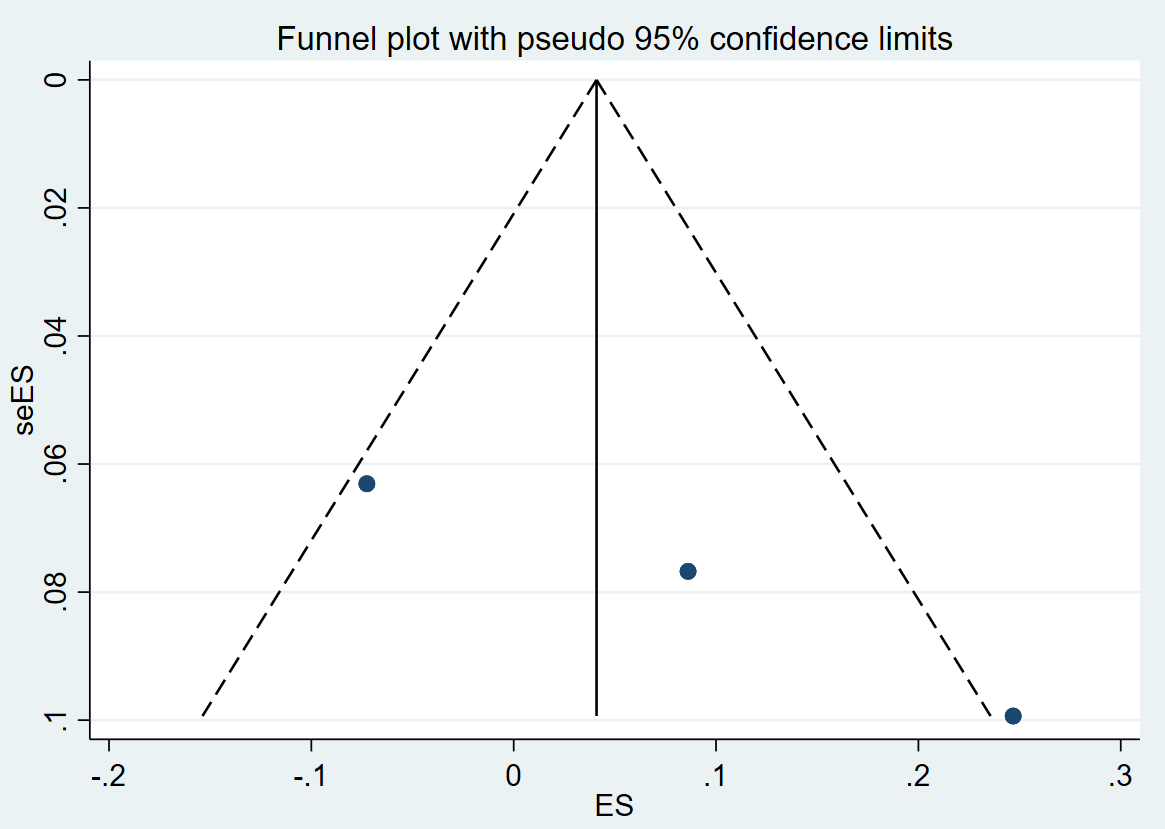 |

| **Racial stratification** | |
| --- | --- |
| White | Black |
| 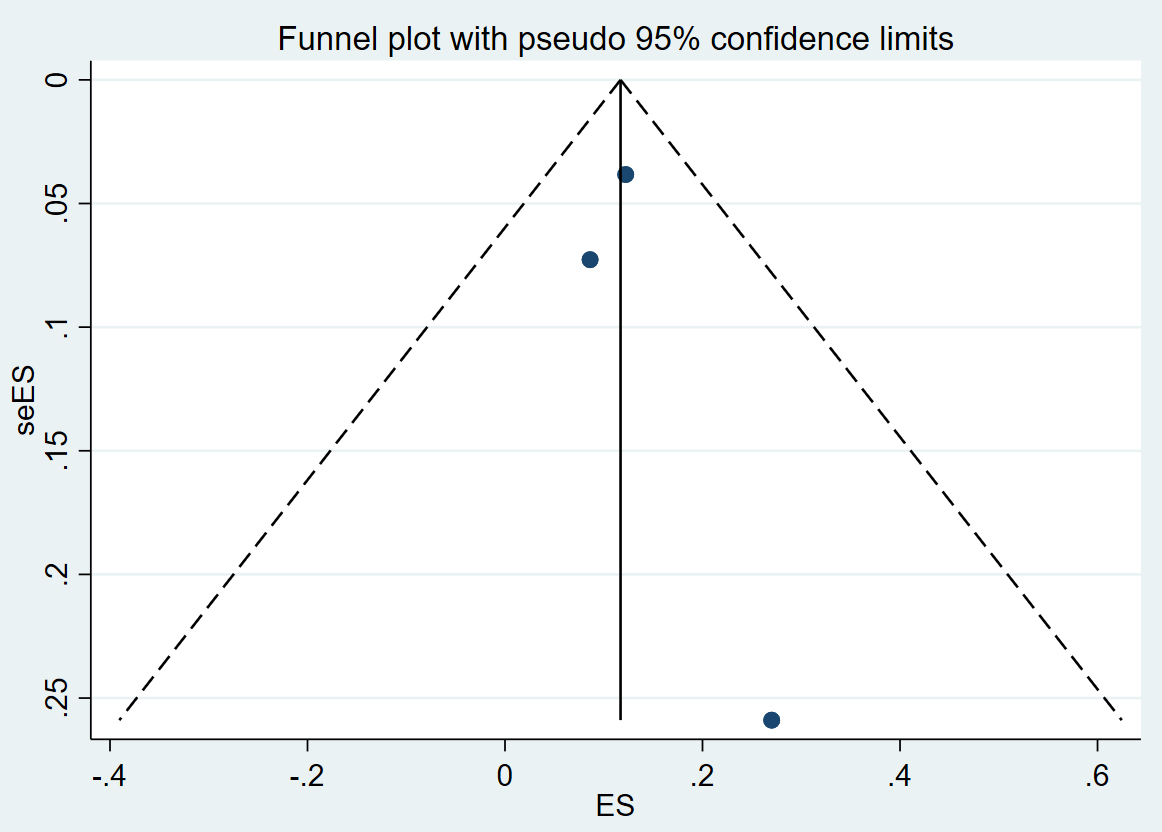 | 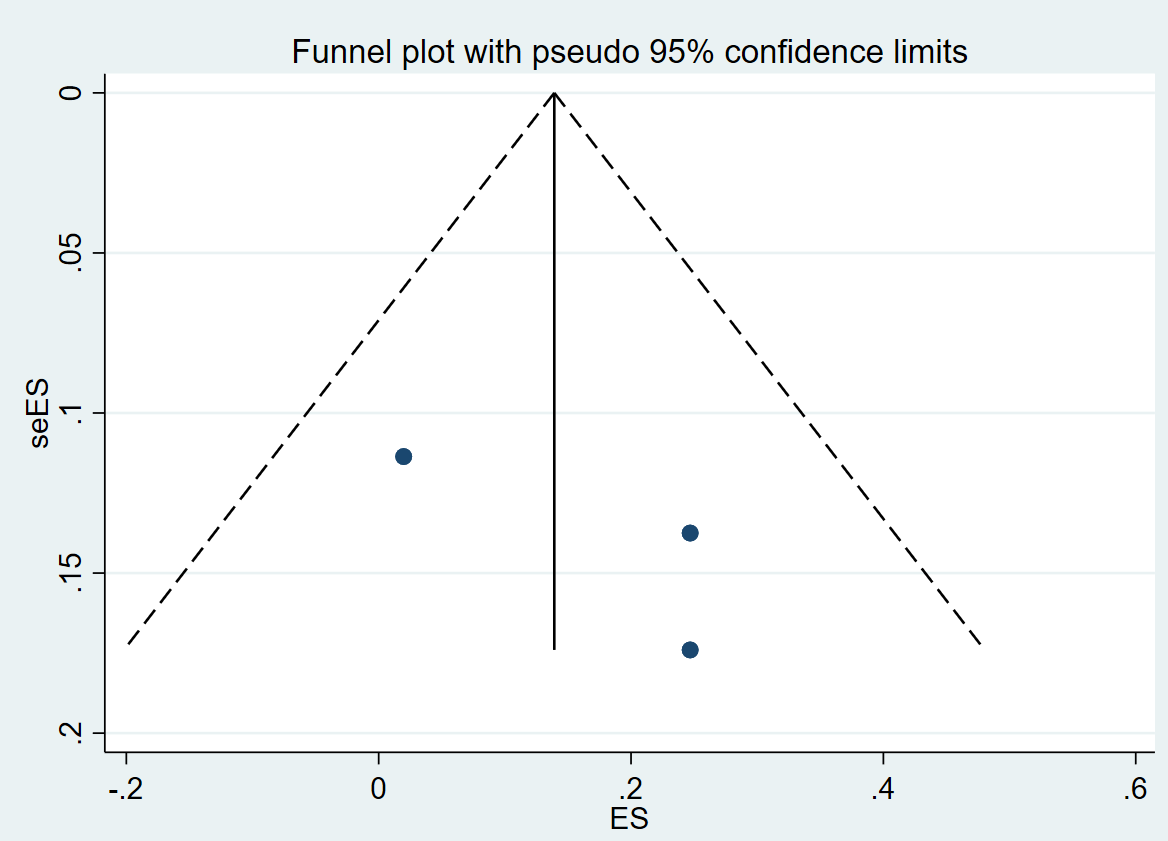 |

**Figure C**: The heterogeneity test results (forest plot) for each subgroup and the p-values for the z-test under two different models.


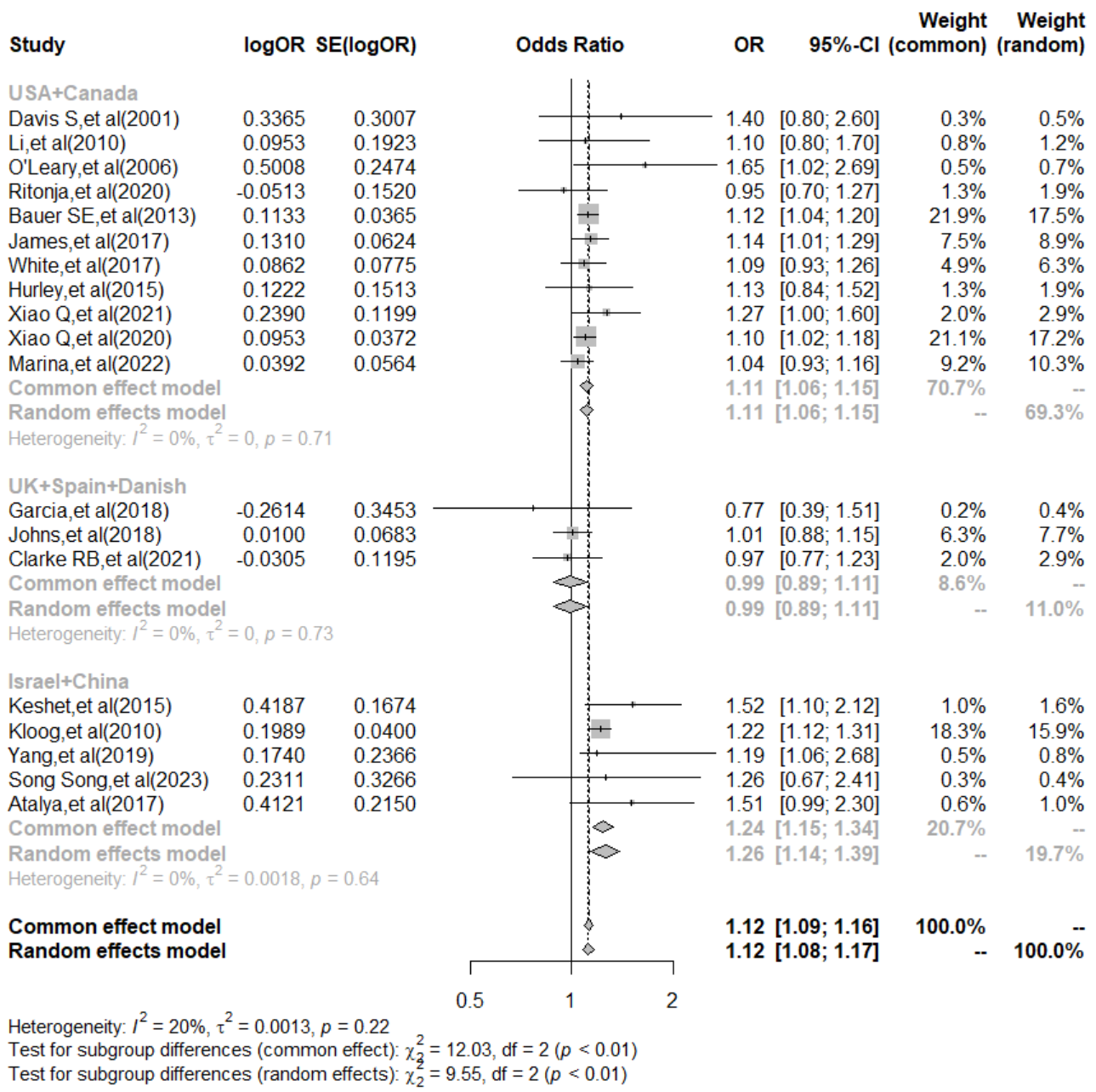


**Continents**


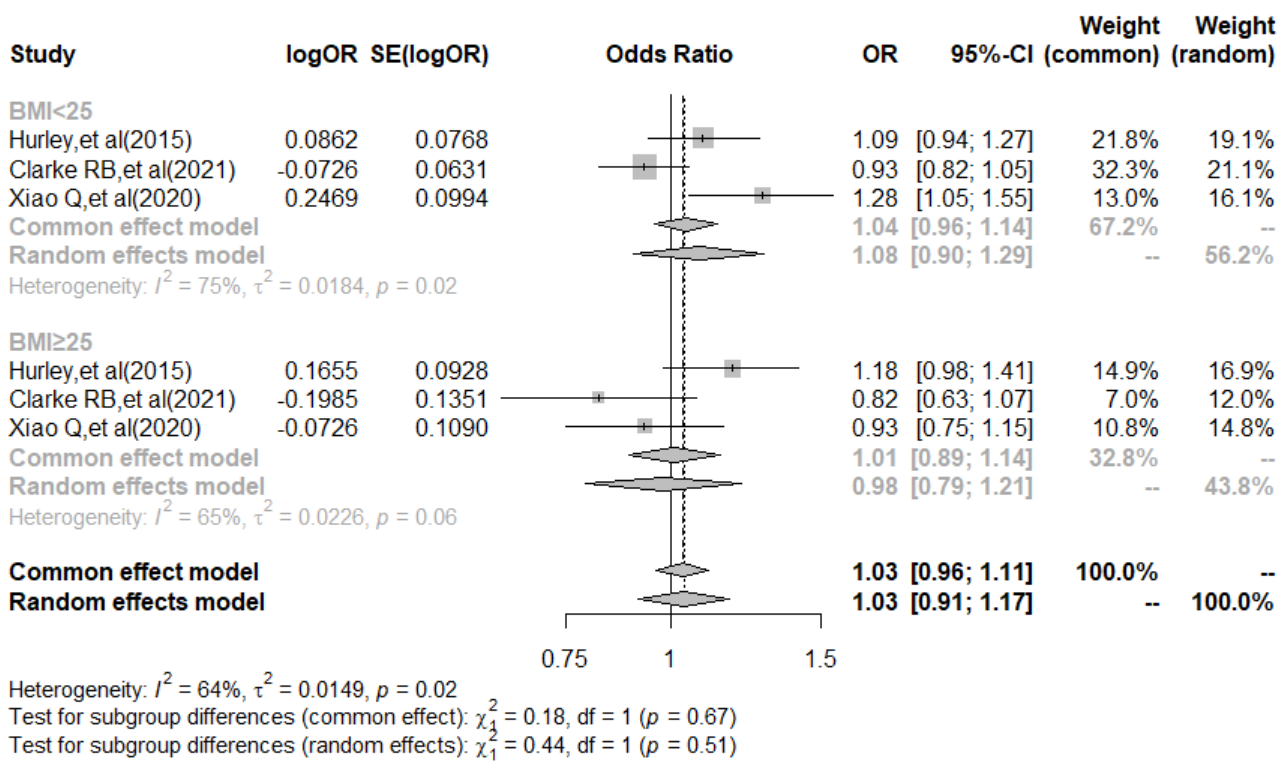


**BMI**


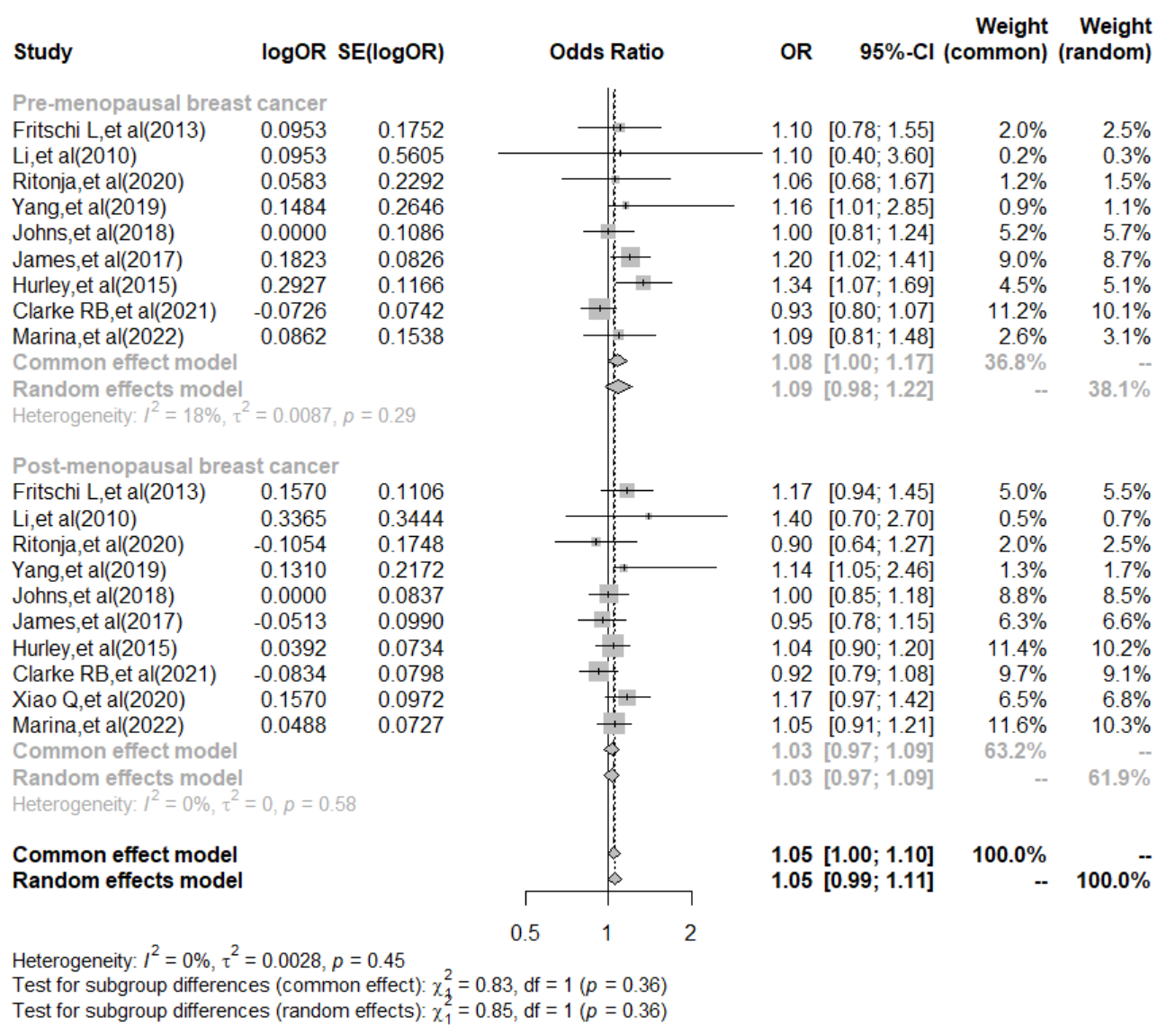


**Menopausal status**

**
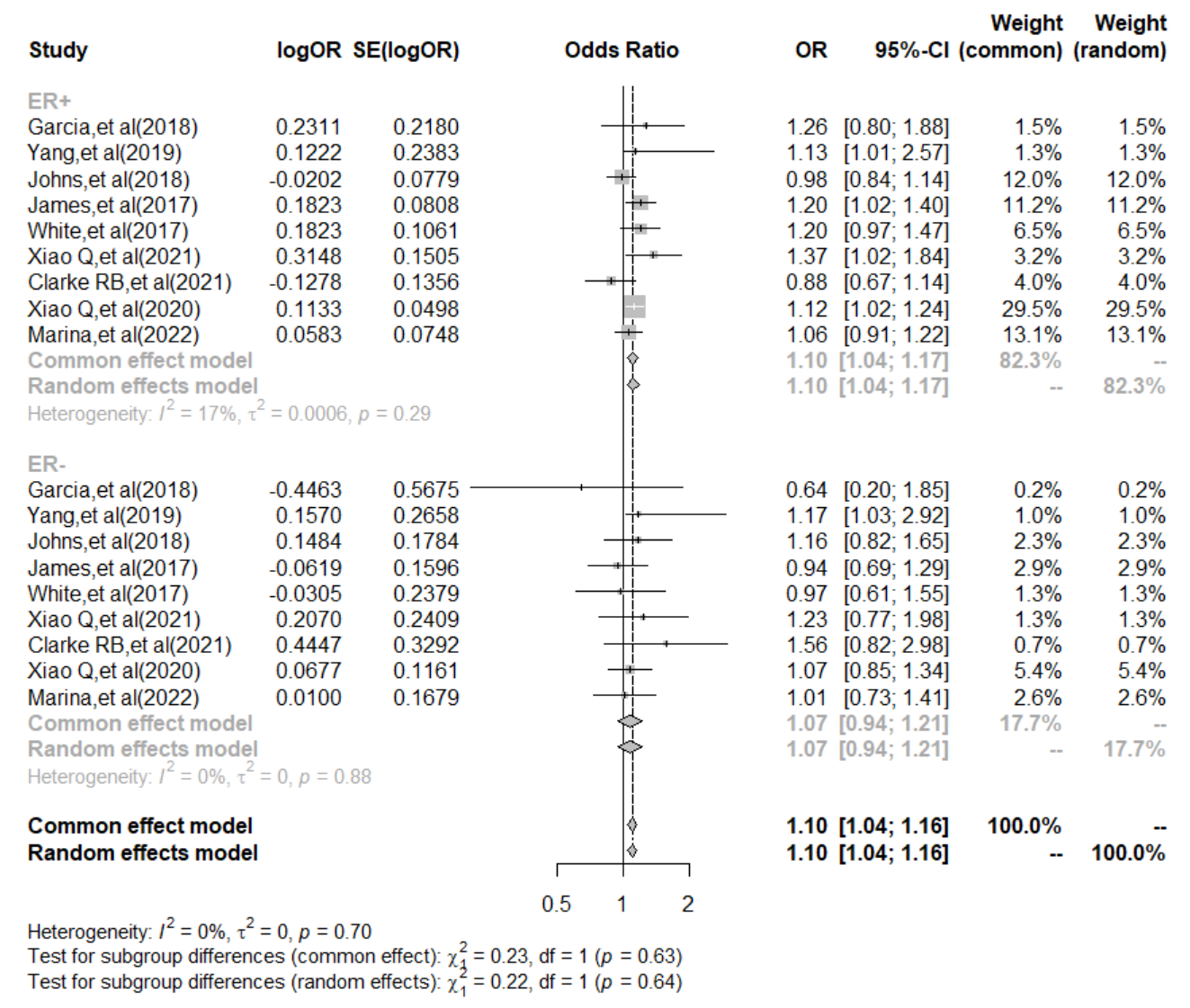
**

**Estrogen receptor status**

**
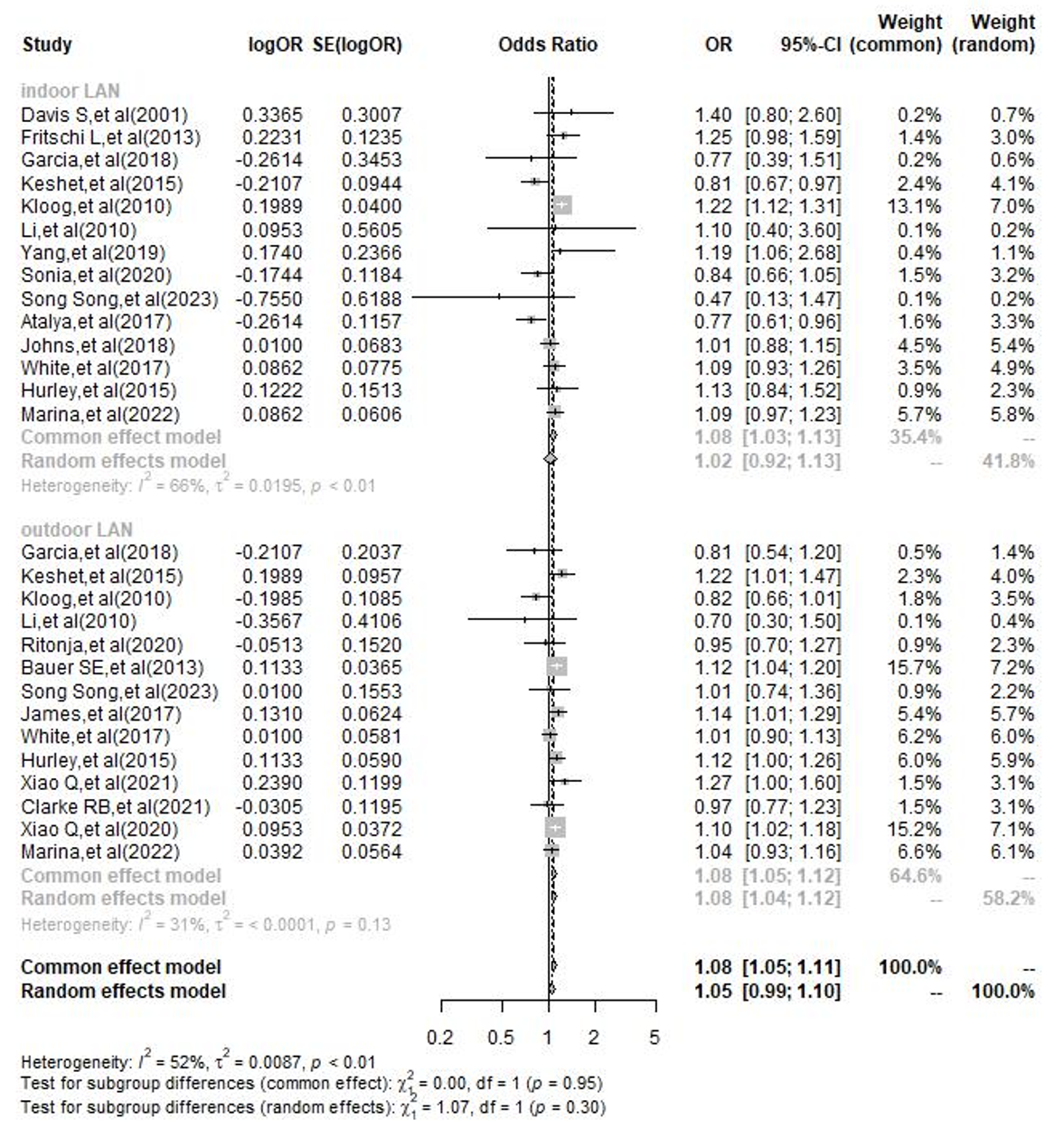
**

**LAN type**

**
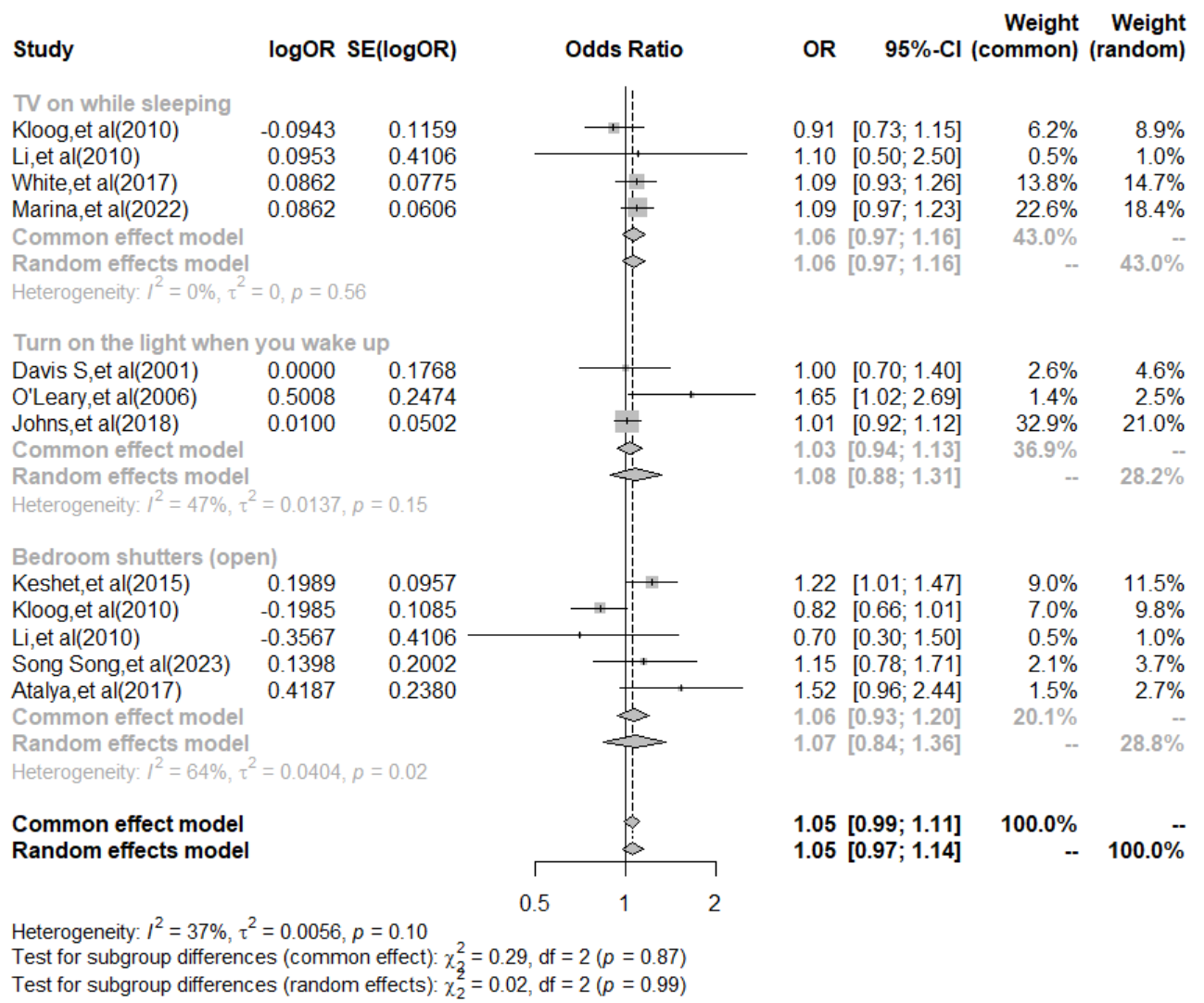
**

**Living habit**

**
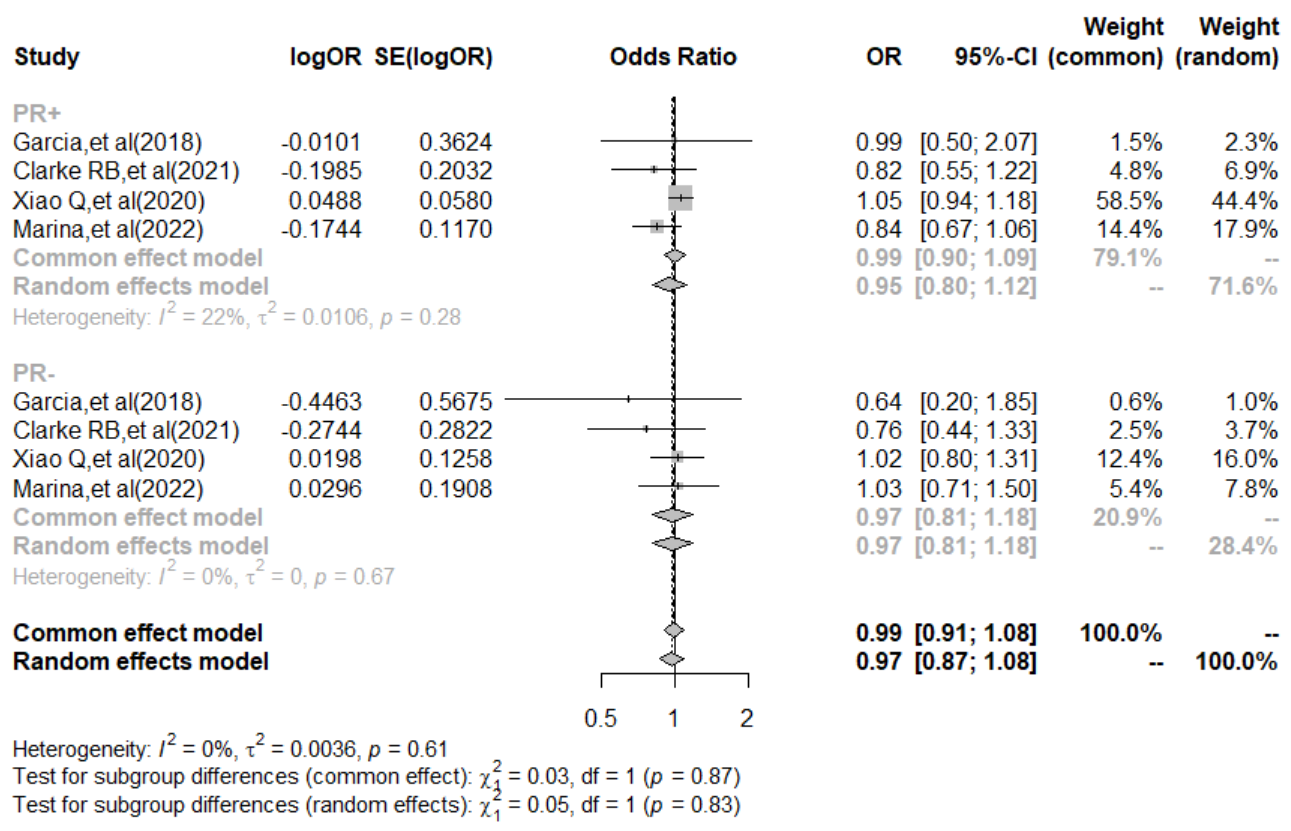
**

**Progesterone receptor status**

**
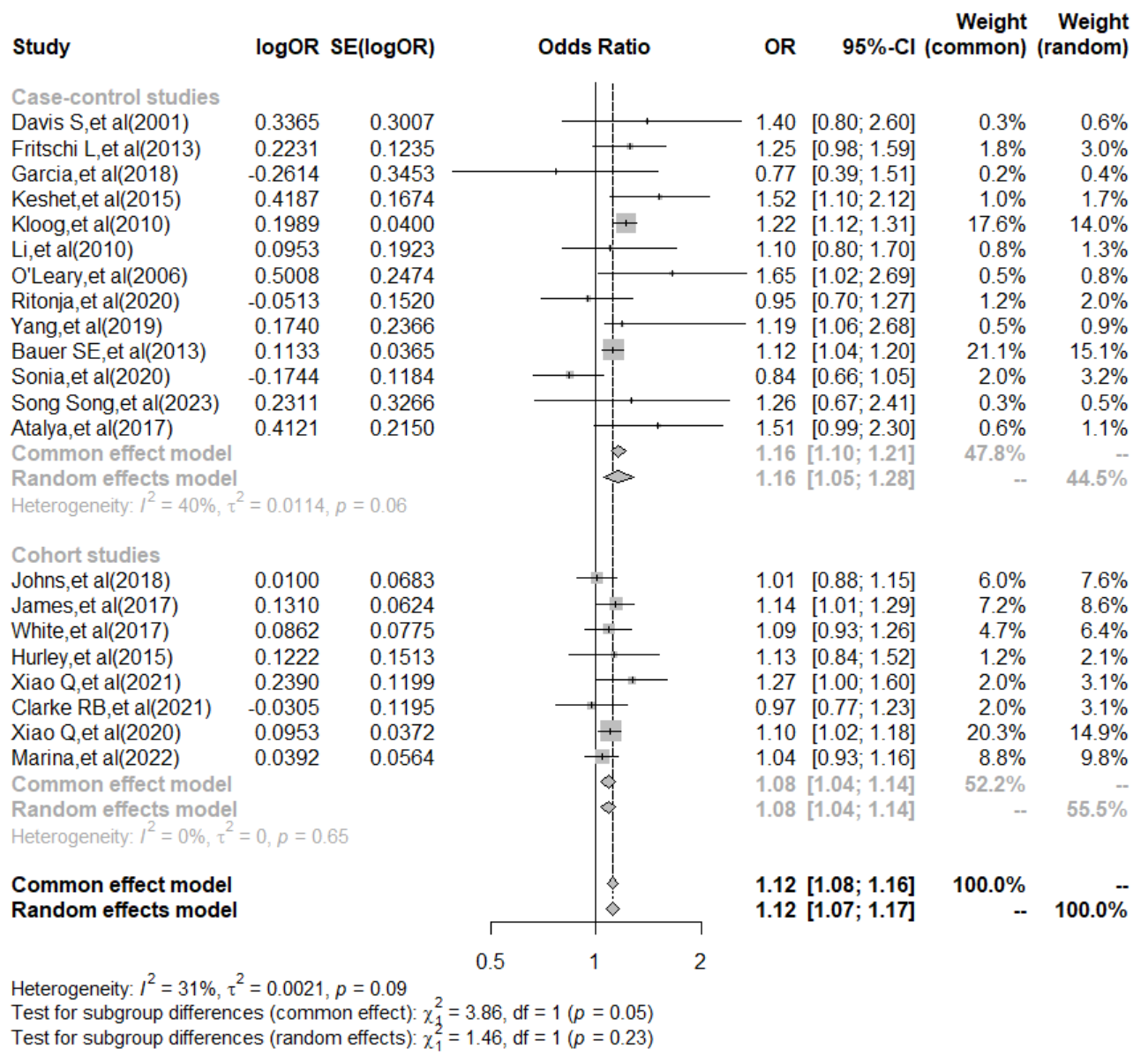
**

**LAN type**

**
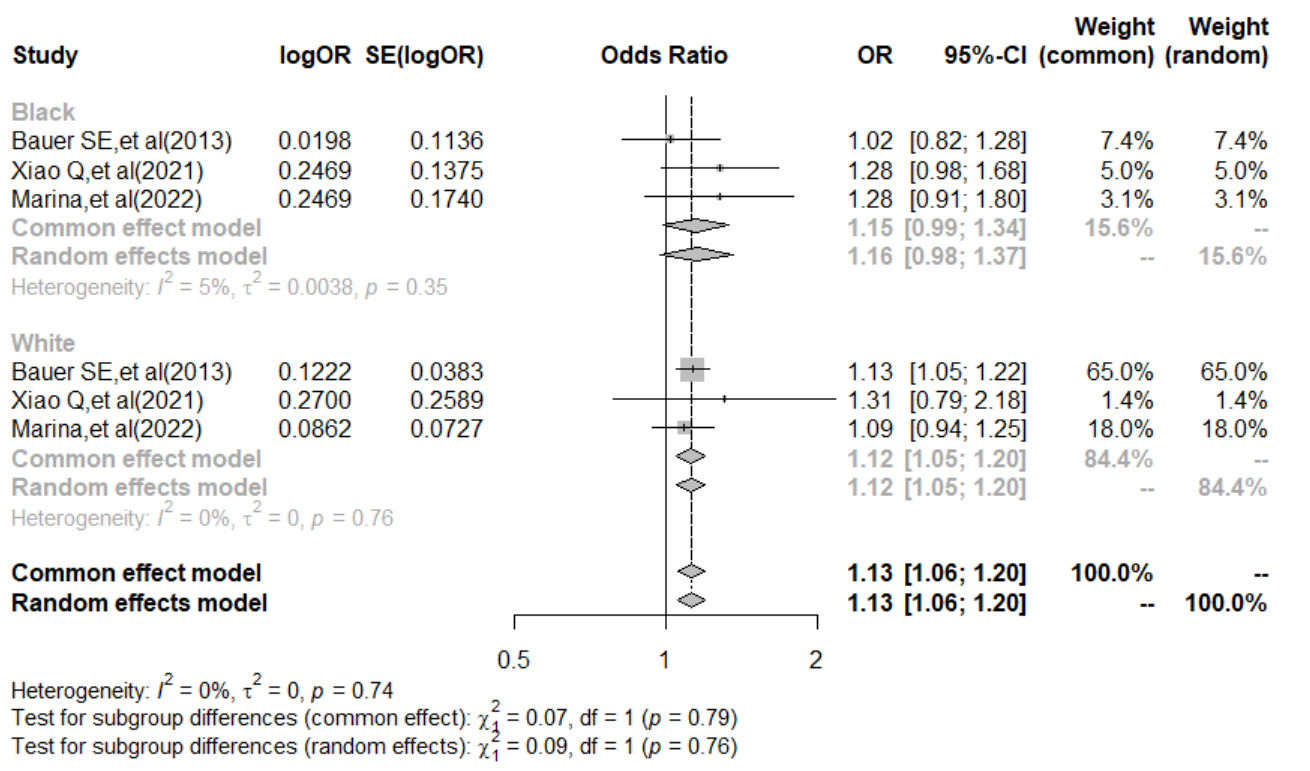
**

**Racial stratification**
